# Leucine Aminopeptidase-Activatable Photosensitizer Enables Enzyme-Responsive Photodynamic Therapy in Neuroblastoma

**DOI:** 10.1021/acsomega.6c01215

**Published:** 2026-06-03

**Authors:** Osman Karaman, Dilay Kepil, Asena Sayin, Mehrdad Forough, Erva Ozkan, Zubeyir Elmazoglu, Gorkem Gunbas

**Affiliations:** † Department of Chemistry, Middle East Technical University, Çankaya, Ankara 06800, Türkiye; ‡ R&D and Innovation Department, Nanografi Nanotechnology, METU Technopolis, Çankaya, Ankara 06531, Türkiye; § Department of Biochemistry, 566936 Ankara Medipol University, Altındağ, Ankara 06050, Türkiye; ∥ Department of Pharmacology, Ankara Medipol University, Altındağ, Ankara 06050, Türkiye

## Abstract

Photodynamic therapy (PDT) is a minimally invasive and tumor-selective treatment modality; however, despite notable progress in several cancer types, effective treatment options for brain tumors remain limited. Although explored only in a limited number of studies, the intrinsic selectivity of photosensitizers bearing activatable groups makes PDT an attractive strategy for brain cancers. Here, we report, for the first time, the photodynamic efficacy of a leucine aminopeptidase (LAP)-activatable iodinated resorufin derivative (**LAP-RI**) in neuroblastoma cells (SH-SY5Y). By incorporating a LAP-responsive handle group, the photosensitizer remains silent until enzymatic activation, exploiting the elevated LAP expression in SH-SY5Y observed in this work. This activatable design enabled a measurable, yet modest (∼2-fold) enhancement in phototoxicity toward neuroblastoma cells relative to healthy fibroblasts, reflecting enzyme-dependent activation rather than strong intrinsic tumor selectivity. Rather than constituting strong intrinsic cell-line selectivity, this difference reflects enzyme-dependent activation, consistent with elevated LAP activity in SH-SY5Y cells. These findings highlight the potential of resorufin-based, enzyme-activatable photosensitizers as a mechanistically selective platform for PDT of extracranial tumors and underscore the broader promise of activatable PDT agents.

## Graphical abstract

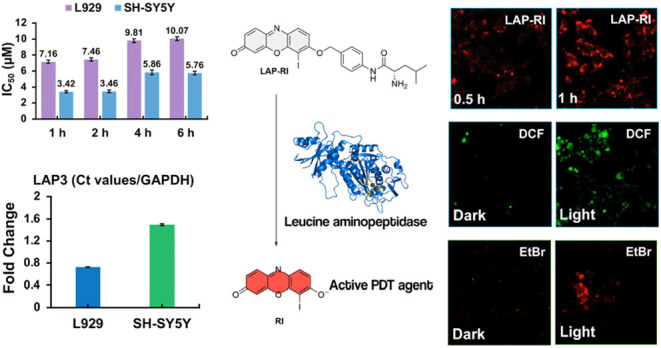

## Introduction

Early and accurate detection, as well as effective treatment, are of the utmost importance for cancer patients, as cancer remains the second leading cause of death worldwide.^1^ Despite significant advancements in cancer prevention, early diagnosis, and treatment during the 21st century, which have led to reduced mortality rates in certain cancer types, conventional diagnostic and therapeutic approaches often fall short in providing precise and efficient solutions for many cancer types, especially biologically aggressive cancers.^2,3^ Three primary factors contribute to the persistent difficulties in treating intra- and extracranial cancers: the high incidence of inoperable tumors due to their location near critical anatomical regions,^4,5^ the limited number of diagnostic and therapeutic agents capable of crossing biological barriers (i.e., blood-brain barrier), the body’s most tightly regulated gateways,^6,7^ and the lack of effective, targeted conventional therapies.^8^

In pediatric oncology, these challenges intersect with the burden of aggressive embryonal tumors such as neuroblastoma (NB).^9^ NB is a malignancy arising from neural-crest-derived cells of the sympathetic nervous system and represents the most common extracranial solid tumor in children under five years of age, accounting for about 10–15% of pediatric cancer deaths.^9^ Current multimodal regimens combining intensive chemotherapy, surgery, radiotherapy, autologous stem cell transplantation, differentiating agents, and immunotherapy have improved 5-year survival for high-risk patients from below 20% to approximately 50%, yet relapse and therapy resistance remain common, and long-term cure rates are still unsatisfactory.^10,11^ This unfavorable outlook, together with substantial late toxicities and the need to preserve quality of life in very young patients, has driven intense efforts to develop more precise, less toxic approaches, including molecularly targeted agents, epigenetic therapies, and next-generation immunotherapies.^11–13^ Given the high mortality and morbidity associated with aggressive extracranial pediatric tumors such as high-risk neuroblastoma and the biological and molecular barriers that limit current treatments, there is an urgent need to develop alternative, targeted strategies that improve tumor specificity while minimizing damage to healthy tissues.

Photodynamic therapy (PDT) has gained significant attention in recent decades due to its minimally invasive nature and its reduced side effects compared to traditional treatment modalities.^14–20^ In conventional PDT, a photosensitizer (PS) in its triplet excited state is generated through the irradiation of the ground-state PS, which undergoes intersystem crossing (ISC) from the singlet to the triplet excited state upon exposure to an appropriate light source. This triplet-state PS then interacts with molecular oxygen (^3^O_2_) to generate reactive oxygen species (ROS), primarily singlet oxygen (^1^O_2_), which is cytotoxic to cells at the site of PDT action.^21–23^ In addition to the advantages of PDT over traditional treatments, enhanced tumor selectivity can be achieved with next-generation PDT agents modified with handle groups to increase treatment efficacy.^24^ A recent and popular approach involves designing activatable photosensitizers (aPSs), which remain inactive in healthy cells even under light exposure and become active exclusively in cancer cells.^25^ This activation occurs upon cleavage of the handle group in response to specific conditions, such as low pH,^26–31^ tumor-associated stimuli,^32–35^ overexpressed biothiols,^36–40^ or enzymes.^41–43^

Leucine aminopeptidase (LAP) is a proteolytic enzyme that catalyzes the hydrolysis of N-terminal leucine residues from proteins and peptides, and its aberrant expression is closely linked to cancer pathogenesis at the molecular level.^44^ In normal cells, LAP expression is typically low, but it is frequently overexpressed in various malignancies, including liver, breast, ovarian, and pancreatic cancers, where it contributes to tumor cell proliferation, invasion, angiogenesis, and drug resistance.^45–48^ Molecular imaging studies using highly selective fluorescent and chemiluminescent probes have demonstrated that LAP activity is significantly elevated in tumor tissues compared to normal tissues, enabling real-time visualization of LAP distribution and activity in living cancer cells and animal models.^46,49–55^ Mechanistically, LAP facilitates the degradation of hydrophobic transmembrane domains of membrane proteins within lysosomes, a process essential for maintaining lysosomal integrity and supporting the high metabolic demands of rapidly proliferating cancer cells; for example, upregulation of lysosomal LAP (LyLAP) in pancreatic ductal adenocarcinoma is necessary for efficient nutrient acquisition via macropinocytosis, and its loss leads to lysosomal dysfunction and impaired tumor growth.^56^ These observations further support the concept that LAP activity is not only a diagnostic marker but may also represent a functional metabolic vulnerability that can be exploited for enzyme-responsive therapeutic activation. Specific isoforms, such as LAP3, have been implicated in tumor progression, metastasis, and poor prognosis across several cancers and are involved in processes such as antigen processing and modulation of the tumor microenvironment.^47,48^ In addition, placental LAP (P-LAP) has been identified as a noninvasive urinary biomarker for invasive ovarian cancer, reflecting its secretion and altered trafficking in malignant cells.^57^ Neuroblastoma cells are characterized by elevated proteolytic activity and increased reliance on amino acid recycling. Therefore, LAP3 activity may facilitate these processes by participating in cytosolic peptide degradation downstream of proteasomal or lysosomal pathways.^58,59^ Through this role, LAP3 may indirectly influence redox balance, signaling cascades, and cell cycle progression, all of which are key determinants of the neuroblastoma cell fate. LAP3 may therefore act as a functional mediator, linking peptide metabolism to tumor cell viability and responsiveness to therapeutic agents. While direct studies on LAP in neuroblastoma are limited, the enzyme’s established roles in tumor cell proliferation, invasion, and resistance mechanisms in other cancers suggest that LAP may similarly contribute to the biology of neuroblastoma and related neural-crest-derived tumors, warranting further investigation.^47,60^

Targeting cancers with LAP-caged photosensitizers represents a promising, selective therapeutic strategy, as these agents are activated by elevated LAP activity in many tumor cells, resulting in localized PDT effects while sparing normal tissues.^46,61–63^ Recent studies have demonstrated that LAP-activatable photosensitizers, such as hemicyanine-based and diketopyrrolopyrrole-based probes, remain nontoxic in the absence of light and are only converted into their active, cytotoxic forms upon enzymatic cleavage by LAP in cancer cells, resulting in effective tumor cell killing with minimal off-target effects. These approaches have demonstrated high selectivity and efficacy across various cancer cell lines and animal models, with negligible toxicity to healthy cells.^62,63^ Thus, the major conceptual advantage of LAP-caged PDT systems is not simply the use of a cytotoxic photosensitizer but the coupling of photosensitizer activation to a tumor-associated enzymatic event, thereby improving molecular selectivity and spatiotemporal control of phototoxicity. Although direct research on LAP-caged photosensitizers in neuroblastoma is limited, the overexpression of LAP in multiple tumor types and the demonstrated success of LAP-activated PDT in other cancers suggest that this strategy could be extended to neuroblastoma and related tumors, offering a new avenue for targeted therapy in which conventional treatments may fall short.^46,61–63^ In this regard, the first LAP-activatable photodynamic therapy (PDT) agent, HCL, was introduced in 2021. This innovative design incorporated leucine into a hemicyanine core via a traceless linker. HCL demonstrated red-shifted absorption and emission upon cleavage, as well as selective photocytotoxicity and a turn-on fluorescence response specifically targeting lung cancer (A549 cell line) and colon cancer (HCT-116 cell line).^61^ Building on this principle, the present study frames LAP-RI as a proof-of-concept, single-trigger enzyme-activatable photosensitizer, using the leucine-caged design to validate LAP-dependent photodynamic activation in neuroblastoma cells. In subsequent molecular designs, the same leucine-responsive activation motif could be adapted to red-shifted or near-infrared photosensitizer scaffolds, such as hemicyanine/cyanine-based dyes, aza-BODIPY systems, or phthalocyanine/chlorin-type chromophores, to improve the photodynamic performance while preserving LAP-dependent enzymatic activation as the central selectivity mechanism.^64–67^

Among the various chromophores explored for photodynamic therapy, resorufin has emerged as a promising scaffold for photosensitizer development, particularly in recent studies, including those from our group, which demonstrates its potential for ROS generation upon structural modification.^62–64^ However, it should be noted that resorufin-based photosensitizers are not yet widely established in the broader PDT field, partly due to inherent photophysical limitations such as absorption at relatively short wavelengths. In this context, iodinated resorufin (RI) has been shown to exhibit enhanced photodynamic activity across multiple cell lines, supporting its utility as a proof-of-concept platform for further optimization.^68–70^ The activatable resorufin core exhibits high cancer selectivity upon cleavage of the targeting group. In this regard, the aim of this study is to demonstrate, for the first time, the efficacy of the well-known PDT agent iodinated resorufin on the neuroblastoma cell line SH-SY5Y by leveraging the high turn-on capability of a LAP-activatable iodinated resorufin. While enzyme-activatable photosensitizers have been explored, their application in neuroblastoma remains largely unreported. In this work, we demonstrate, for the first time, LAP-activatable photodynamic therapy in neuroblastoma using an iodinated resorufin scaffold. Importantly, rather than focusing on maximizing selectivity, we establish a quantitative relationship between LAP expression and photodynamic response, providing a mechanistic framework for enzyme-limited activation in a minimal single-trigger system. Incorporating a LAP-activatable moiety into photosensitizers is a promising strategy for achieving tumor selectivity, as LAP is frequently overexpressed in cancer cells compared to healthy tissues, enabling selective activation and minimizing off-target toxicity.^71,72^ While LAP-activated photosensitizers have shown high selectivity and potent photocytotoxicity in other cancer models, their application in neuroblastoma and brain tumors remains largely unexplored.^61,63^ This study addresses this gap by presenting the first evidence of LAP-activatable photodynamic action in neuroblastoma, achieving a 2-fold selectivity for neuroblastoma cells (IC_50_ = 3.42 μM) over healthy fibroblast cells (IC_50_ = 7.16 μM). **LAP-RI** displayed red-shifted absorption and emission upon cleavage when illuminated with 595 nm LED light. These findings indicate that **LAP-RI** is a promising LAP-sensitive theranostic photosensitizer with potential for use as an image-guided, tumor-selective PDT agent for treating cancers with elevated LAP expression, underscoring the potential of enzyme-activatable photosensitizers to enhance the precision and efficacy of PDT for brain tumors, supporting the broader trend in the literature toward targeted, tumor-microenvironment-responsive therapies for improved cancer treatment outcomes.

## Results and Discussion

For the synthesis of LAP-activatable resorufin-based PS, **LAP-RI**, first, boc-protected leucine was coupled with self-immolative linker^73–75^ *p*-aminobenzyl alcohol in the presence of 2-ethoxy-1-ethoxycarbonyl-1,2-dihydroquinoline (EEDQ) to give compound **1** with a high yield. The Appel reaction afforded compound **2** in a moderate yield, which underwent an S_N_2 reaction with the commercially available resorufin sodium salt. Compound **3** was iodinated with molecular iodine in the presence of iodic acid, affording compound **4** in a 68% yield. Finally, the Boc protection was removed, yielding the target PS **LAP-RI**. Prior to investigating its photophysical properties and conducting *in vitro* cell studies, **LAP-RI** was purified by reversed-phase HPLC to achieve greater than 95% purity and was characterized by ^1^H and ^13^C NMR and HRMS. (Figures S2–S11). Overall, the target PS, **LAP-RI**, was synthesized with a 14.5% yield over 5 steps (Scheme 1).^70^

**Scheme 1:**
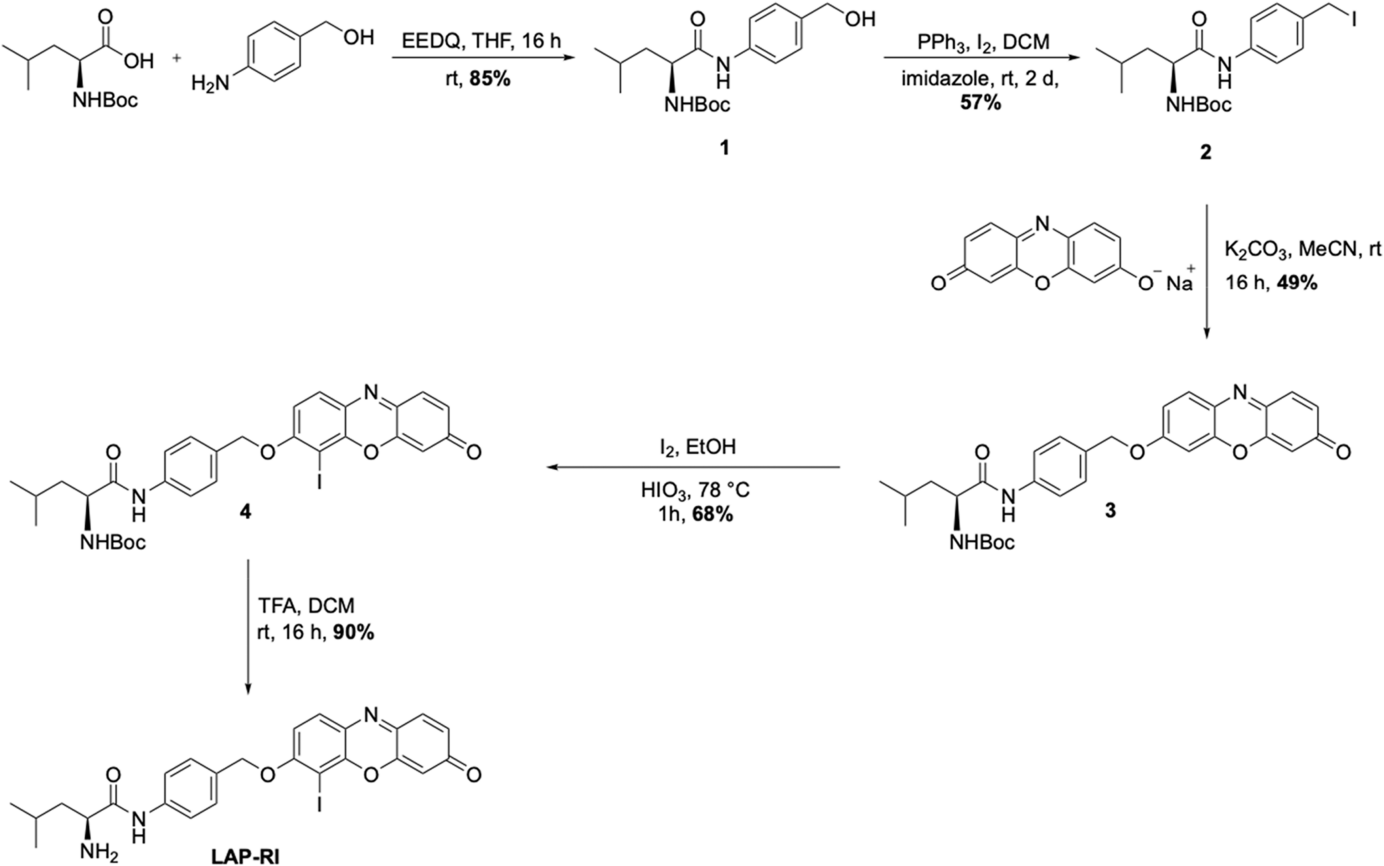
Synthetic Route for **LAP-RI**

**LAP-RI** exhibited two distinct absorption maxima centered at 410 and 490 nm, since the caged agent’s electron delocalization is hindered. The concentration-dependent activation of **LAP-RI** was then investigated. **LAP-RI** (10 μM in PBS, 1% DMSO, pH 7.4) was incubated with LAP enzyme at concentrations ranging from 0 to 5 units. After 95 min of incubation at 37 °C, enzyme-mediated conversion of **LAP-RI** to **RI** was observed, evidenced by the progressive diminishing of the initial absorption bands and the emergence of the characteristic **RI** absorption band at 580 nm, which correlated with increasing enzyme concentration (Figure 1a). Time-dependent release of **RI** in the presence of 0.3 LAP units over 210 min has also been demonstrated (Figure 1b). The fluorescence quantum yield, photostability, and singlet-oxygen-generating capacity of **RI** were evaluated in our previous work.^53^ The Φ_F_ of **RI** was calculated to be 6.8%, and the Φ_Δ_ of **RI** was calculated as 54%.^53–55^

**Figure 1:**
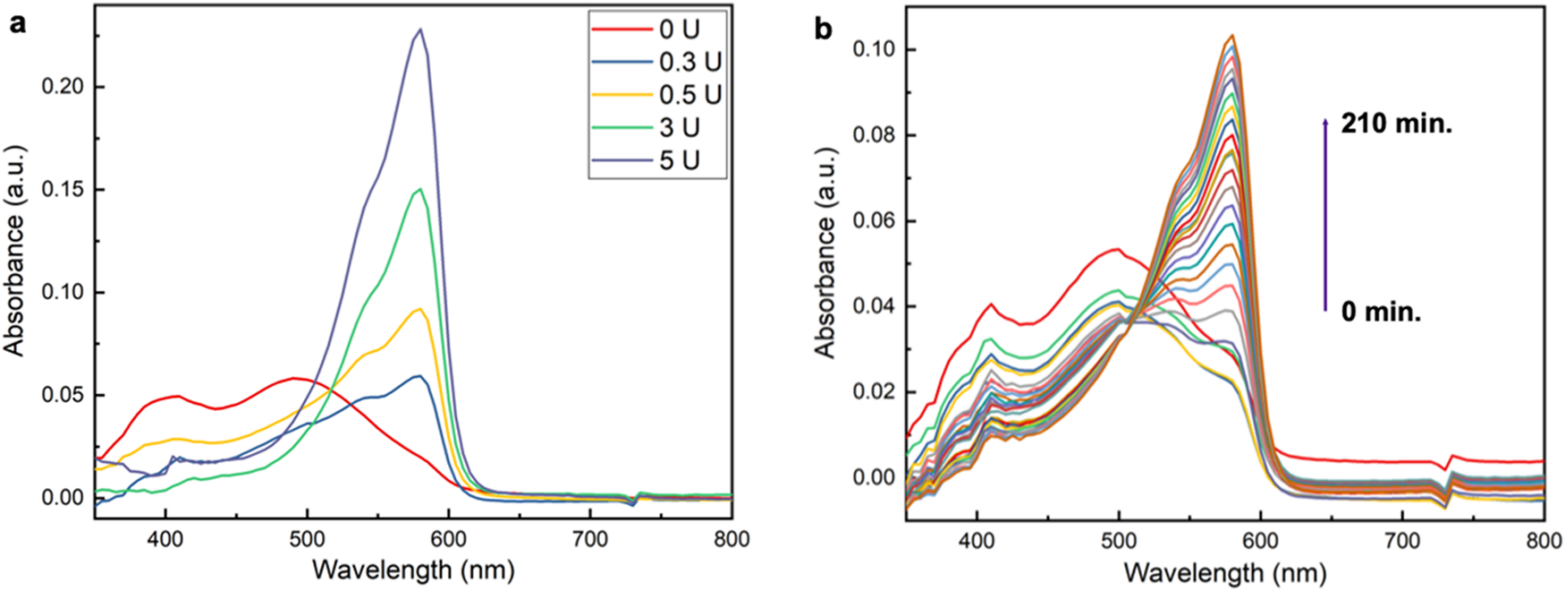
(a) LAP concentration-dependent UV–vis spectra changes of 10 μM **LAP-RI** following 95 min incubation at 37 °C. (b) Time-dependent release of **RI** with 0.3 units LAP from 0 to 210 min.

The potential effect of **LAP-RI** as a PDT agent was investigated *in vitro*, using SH-SY5Y neuroblastoma cells and L929 healthy fibroblast cells. Cell viability was assessed using the MTT assay following exposure to increasing concentrations of **LAP-RI** (0–10 μM). As shown in Figure 2a, incubation of both cell lines with **LAP-RI** for 24 h under dark conditions did not result in a significant reduction in cell viability, confirming that **LAP-RI** exhibits negligible dark cytotoxicity (*n* = 6). Subsequently, the photodynamic activity of **LAP-RI** was examined by incubating cells with **LAP-RI** for varying durations (1, 2, 4, and 6 h) in the dark, followed by LED irradiation (595 nm, 8.12 mW/cm^2^) for 2 h and a 24 h recovery period under dark conditions. Light dose is higher compared to averages in PDT literature, but this irradiation condition is comparable to previously reported *in vitro* PDT protocols in glioma/glioblastoma models, including LED-based PDT in human glioma cells (40–60 J/cm^2^) and THPTS-mediated PDT in glioblastoma models (20–30 J/cm^2^).^77–79^ It should also be noted that the absorption profile of resorufin (580–595 nm) lies below the commonly accepted therapeutic optical window (>630 nm), where light penetration in biological tissues is more favorable. In the present study, resorufin was selected as a well-established, synthetically accessible photosensitizer scaffold that enables efficient ROS generation upon iodination and facilitates the incorporation of enzyme-responsive functionality. Therefore, despite its suboptimal absorption wavelength for deep tissue applications, the current system remains well-suited for *in vitro* validation and mechanistic investigation of enzyme-responsive photodynamic activation.^80^ Accordingly, this system should be considered as a proof-of-concept activatable platform rather than a clinically optimized phototherapeutic agent. Future work will focus on extending this enzyme-activatable design to red-shifted and near-infrared chromophores to improve tissue penetration and translational potential.

**Figure 2:**
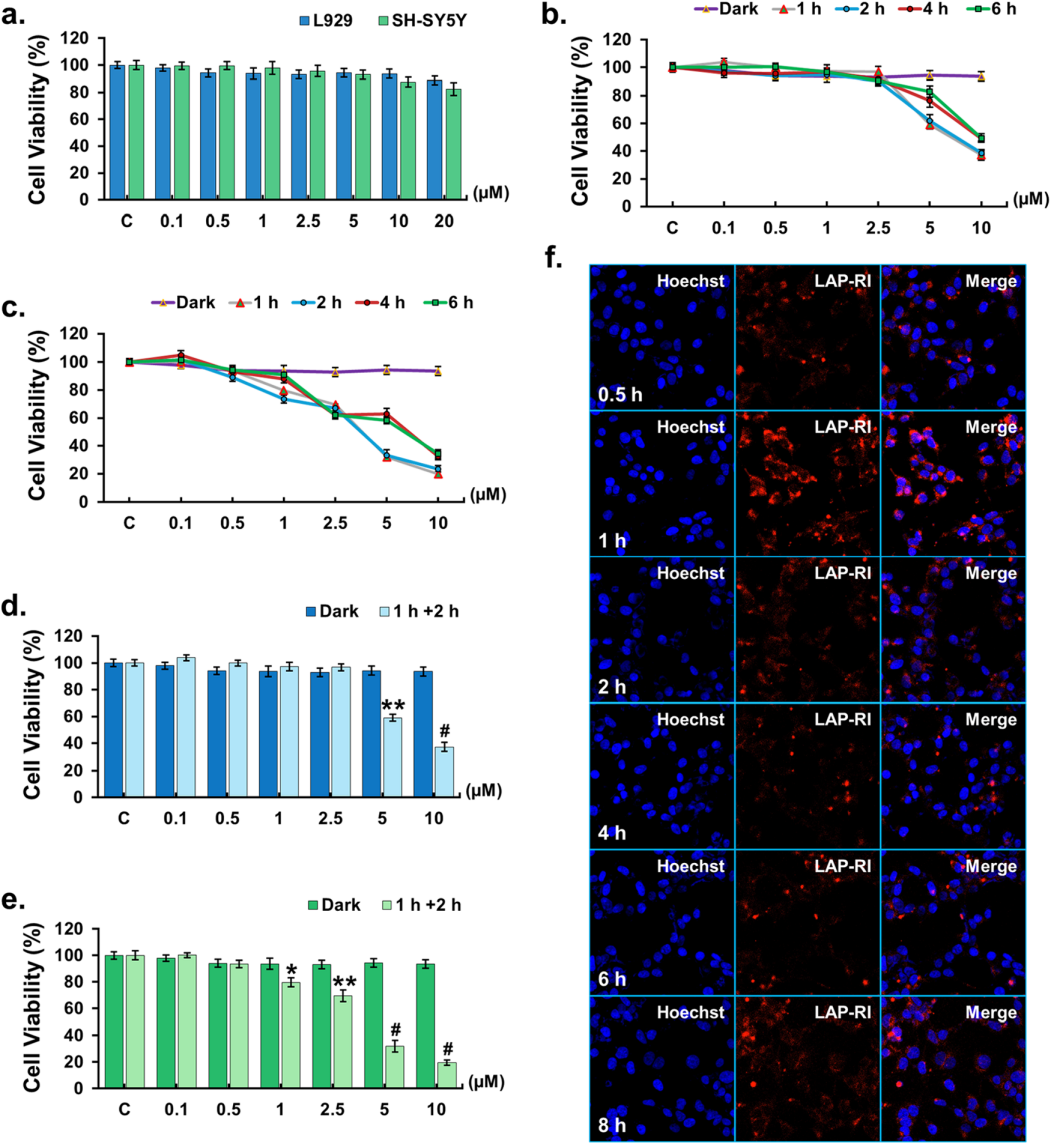
(a) Dark cytotoxicity of **LAP-RI** following 24 h exposure in L929 and SH-SY5Y cells, demonstrating minimal intrinsic toxicity in the absence of light activation (*n* = 6). (b, c) Time- and concentration-dependent phototoxicity of **LAP-RI** (1–10 μM) in L929 and SH-SY5Y cells after incubation for 1, 2, 4, or 6 h, followed by 2 h LED (595 nm, 8.12 mW/cm^2^) irradiation, and a 24 h recovery period in the dark (*n* = 6). (d, e) Concentration-dependent phototoxic responses in L929 and SH-SY5Y cells after 1 h preincubation with **LAP-RI**, followed by 2 h LED irradiation, and 24 h recovery (*n* = 6). (f) Confocal microscopy images demonstrating time-dependent intracellular activation and accumulation of **LAP-RI** (1 μM) in SH-SY5Y cells (*n* = 4). Blue: Hoechst 33342 (nuclei); red: **LAP-RI**. Scale bar: 10 μm. *p < 0.05, **p < 0.01, ^#^ *p* < 0.001 vs dark controls.

Upon light exposure, **LAP-RI** induced pronounced phototoxic effects in a time- and concentration-dependent manner in both cell lines (Figure 2b,c). However, SH-SY5Y neuroblastoma cells exhibited significantly greater sensitivity to photoactivated **LAP-RI** compared to L929 fibroblasts. Increasing incubation times before LED irradiation resulted in progressively greater phototoxicity, indicating a strong dependence of **LAP-RI**-induced toxicity on the duration of preirradiation exposure. Furthermore, when the data obtained after 1 h of **LAP-RI** incubation were compared with the corresponding dark controls, no significant cytotoxicity was observed in L929 fibroblast cells at concentrations up to 2.5 μM. In contrast, SH-SY5Y neuroblastoma cells exhibited a substantial reduction in cell viability at 2.5 μM under identical irradiation conditions, indicating an early and selective phototoxic response (Figure 2d,e).

Quantitative analysis revealed an IC_50_ value of 3.42 ± 0.14 μM for SH-SY5Y cells, whereas L929 fibroblasts displayed substantially lower phototoxic sensitivity, with an IC_50_ value of 7.16 ± 0.21 μM after 1 h incubation. This approximately 2-fold difference reflects a modest but reproducible enhancement in phototoxic response toward neuroblastoma cells. This approximately 2-fold difference reflects a modest but reproducible enhancement in the phototoxic response toward neuroblastoma cells, which should be interpreted as enzyme-dependent activation under simplified *in vitro* conditions rather than as evidence of strong intrinsic tumor selectivity.

Differences in cellular uptake, metabolic activity, proliferation rate, and intrinsic photosensitivity are well-known to influence PDT outcomes across cell lines. The enhanced response in SH-SY5Y cells, together with their elevated LAP3 expression and time-dependent intracellular activation, supports an activation-driven mechanism rather than nonspecific photosensitivity. Consistent with this interpretation, confocal microscopy revealed a time-dependent intracellular activation profile of **LAP-RI** (1 μM) in SH-SY5Y cells (Figure 2f). Maximal intracellular fluorescence was observed after 1 h of incubation, whereas prolonged incubation (2, 4, and 6 h) resulted in reduced fluorescence intensity. This activation profile closely paralleled the cytotoxicity data, with the lowest IC_50_ value also observed at the 1 h incubation time (Figure 3a), indicating that **LAP-RI** exhibits optimal photoactivation and photodynamic efficacy under short preincubation conditions.

**Figure 3:**
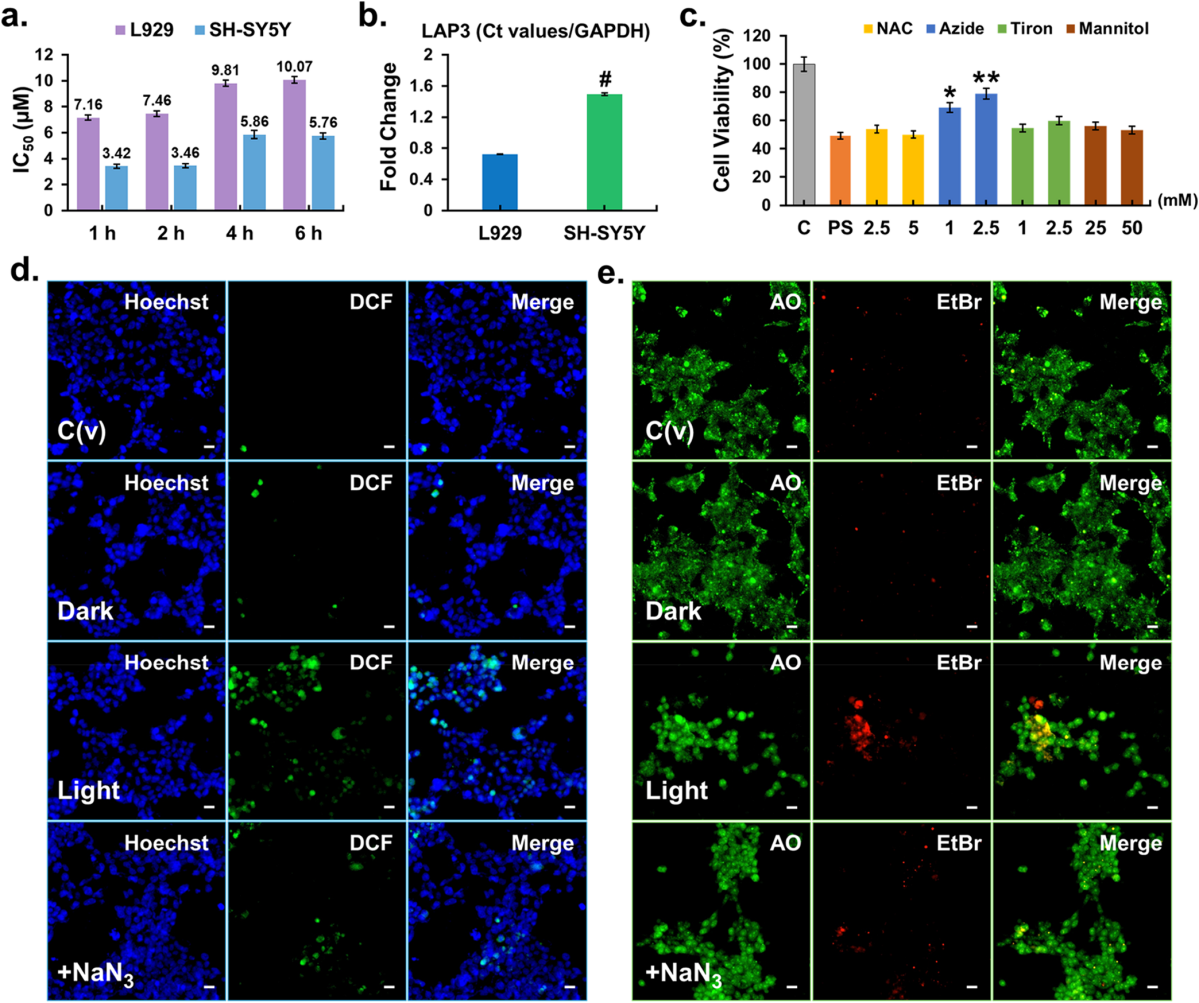
(a) Time-dependent half-maximal inhibitory concentration (IC_50_) values of **LAP-RI** following PDT application, determined after varying preincubation periods prior to LED irradiation (*n* = 6). (b) Relative expression levels of the LAP3 gene in L929 and SH-SY5Y cells, quantified by RT-qPCR and normalized to the housekeeping gene GAPDH (*n* = 3). (c) ROS scavenger assays evaluating the contribution of specific ROS species to **LAP-RI**-mediated phototoxicity (*n* = 6). (d) Intracellular ROS generation measured by the DCFH-DA assay after **LAP-RI** treatment (in IC_50_ concentration, 1 h) under dark and light conditions in the presence or absence of NaN_3_. (e) Acridine orange/ethidium bromide (AO/EtBr) dual-staining after **LAP-RI**-mediated PDT application in SH-SY5Y cells (*n* = 6). PDT protocol, distinguishing viable (green), late apoptotic (yellow-orange), and necrotic (red) cell populations. Scale bar: 20 μm. *p < 0.05, **p < 0.01, ^#^ *p* < 0.001 vs L929 or PS.

To further elucidate the photodynamic behavior and mechanism of action of **LAP-RI**, additional analyses were performed focusing on time-dependent potency, ROS involvement, and the mode of cell death following photodynamic treatment. The time-dependent IC_50_ values of **LAP-RI** were determined after varying preincubation durations before LED irradiation. As shown in Figure 3a, increasing the incubation time before light exposure resulted in progressively higher IC_50_ values, indicating reduced photodynamic potency at longer preincubation durations. These results demonstrate that the photodynamic efficacy of **LAP-RI** is strongly dependent on incubation time, with maximal activity observed at shorter preirradiation exposure periods. Notably, across all incubation times tested, L929 fibroblast cells consistently exhibited higher IC_50_ values than SH-SY5Y neuroblastoma cells, indicating reduced phototoxicity and further supporting the enzyme-dependent enhancement of **LAP-RI**’s photodynamic effect on neuroblastoma cells. To determine whether this differential cellular responsiveness was associated with LAP3 levels, the relative LAP3 gene expression was quantified in both cell lines using RT-qPCR. As illustrated in Figure 3b, a 2-fold increase in LAP3 gene expression was observed in SH-SY5Y cells, which may partially contribute to the enhanced phototoxic response following **LAP-RI** activation, consistent with an enzyme-mediated rather than inherently selective cytotoxic mechanism. Recent work on enzyme-responsive PDT indicates that such single-trigger systems, which rely on a single enzymatic cue without added targeting, nanoassembly, or logic-gate amplification, typically yield modest but statistically reproducible differences in IC_50_ that quantitatively mirror the underlying enzyme-expression gradient.^71,81^ In contrast, the higher selectivity indices frequently reported in the PDT literature generally arise from multilayered activation architectures, such as double-locked photodynamic molecular beacons that require sequential cleavage by MMP-2 and cathepsin B, or extrinsic-enzyme and FRET-gated constructs, which routinely show 3–6-fold differences in fluorescence activation and photocytotoxicity between permissive and nonpermissive conditions by coupling enzyme action to quenching/dequenching and multistep amplification.^80,82^ Enzyme-activatable nanoplatforms that integrate protease responsiveness with nanoformulation-driven retention, self-oxygenation, or subcellular targeting likewise achieve higher tumor-versus-normal selectivity by amplifying intracellular photosensitizer accumulation rather than relying solely on a single protease gradient.^83,84^ In this context, the ∼2-fold LAP3 upregulation observed here is biologically interpretable as a quantitative, proof-of-principle readout of enzyme-limited activation in a minimal LAP-responsive construct. In this context, the present system is intentionally designed as a minimal, single-trigger platform applied to a previously unexplored neuroblastoma model. The observed ∼2-fold selectivity is therefore not a limitation but a direct reflection of enzyme-dependent activation, providing a mechanistically interpretable basis for future optimization toward more advanced, highly selective systems.

The role of ROS in **LAP-RI**-mediated phototoxicity was investigated using a panel of ROS scavengers. As shown in Figure 3c, the presence of specific scavengers significantly attenuated **LAP-RI**-induced phototoxicity, indicating that ROS play a central role in the observed cell death following light activation. In particular, the marked protective effect observed with sodium azide (NaN_3_), a known singlet oxygen scavenger, suggests a substantial contribution of singlet oxygen to **LAP-RI**-mediated PDT efficacy. In line with these findings, the intracellular ROS generation was further examined using the cell-permeable probe 2′,7′-dichlorofluorescein diacetate (DCFH_2_-DA). Following intracellular hydrolysis by esterases, oxidation of the probe by ROS results in bright green fluorescence. As shown in Figure 3d, SH-SY5Y cells treated with **LAP-RI** at the IC_50_ concentration (1 h incubation) and exposed to LED irradiation exhibited a pronounced increase in DCF fluorescence compared to dark controls, confirming light-dependent ROS generation. In contrast, cotreatment with NaN_3_ markedly reduced fluorescence intensity, further corroborating the involvement of ROS in **LAP-RI**-induced phototoxicity and aligning with the scavenger assay and viability data.

Finally, the mode of cell death induced by **LAP-RI**-mediated PDT was assessed by using acridine orange/ethidium bromide (AO/EtBr) dual staining. As shown in Figure 3e, untreated control cells predominantly exhibited green fluorescence, indicating viability. In contrast, SH-SY5Y cells treated with **LAP-RI** and subjected to LED irradiation displayed a yellow-orange fluorescence in merged images, characteristic of apoptosis-associated changes in membrane permeability. Red fluorescence, indicative of necrosis, was minimal under the conditions. Notably, the addition of NaN_3_ substantially reduced the apoptotic and necrotic cell populations, further confirming that ROS generation is a key mediator of **LAP-RI**-induced cytotoxicity. Collectively, these results demonstrate that **LAP-RI**-mediated photodynamic therapy induces a time-dependent increase in potency, primarily via ROS generation, leading predominantly to apoptotic cell death in neuroblastoma cells. The strong inhibitory effect of ROS scavengers, combined with direct intracellular ROS detection and apoptosis-specific staining, provides compelling evidence that **LAP-RI** exerts its phototoxic effects via ROS-dependent pathways, reinforcing its potential as a selective and promising PDT agent.

## Conclusion

In conclusion, the first LAP-activatable iodinated resorufin was successfully realized via a five-step synthesis process. This photosensitizer exhibits a pronounced turn-on response in both absorption and emission profiles and efficient singlet oxygen generation in aqueous media upon enzymatic cleavage of its LAP-responsive handle group. The photodynamic potential of **LAP-RI** was evaluated *in vitro* using neuroblastoma and healthy fibroblast cell lines, with negligible dark toxicity observed in both cell types. Upon LED irradiation, **LAP-RI** induced a modest but reproducible enhancement in the phototoxic response in SH-SY5Y cells compared to L929 fibroblasts. This differential response arises from enzyme-dependent activation rather than large intrinsic differences in cytotoxic sensitivity, consistent with the expected behavior of single-trigger activatable photosensitizers under simplified *in vitro* conditions. Maximal photodynamic efficacy was observed at shorter preincubation times, corresponding to the lowest IC_50_ values and peak intracellular activation, whereas prolonged incubation led to reduced photoactivation and potency. While this inverse time-potency relationship is consistent with optimal enzymatic uncaging, we acknowledge that alternative factors such as **LAP-RI** degradation, efflux, or photophysical quenching cannot be excluded in the absence of dedicated uptake and mechanistic experiments. Importantly, **LAP-RI**-mediated phototoxicity was accompanied by a significant increase in intracellular ROS generation, which was markedly suppressed in the presence of singlet oxygen scavenger NaN_3_, confirming a ROS-dependent mechanism of action. Furthermore, cell death analysis revealed that **LAP-RI** induces apoptotic cell death in the neuroblastoma cells.

The higher photodynamic responsiveness of SH-SY5Y cells was further associated with elevated LAP3 gene expression, supporting the enzyme-dependent activation of **LAP-RI** rather than definitive tumor-intrinsic selectivity. Collectively, these findings establish **LAP-RI** as an effective, enzyme-activatable photosensitizer that combines ROS-mediated apoptosis with modest enzyme-limited differential responsiveness. Accordingly, **LAP-RI** should be interpreted as a proof-of-principle enzyme-activatable photosensitizer, highlighting its utility as a controllable molecular platform rather than a fully optimized therapeutic agent. This work underscores the critical role of activatable agents in advancing the photodynamic strategies for extracranial tumor treatment. Taken together, this study emphasizes that the value of **LAP-RI** lies not in a large IC_50_ separation between cell lines but in its controllable, enzyme-responsive activation mechanism, which provides a rational molecular basis for improving spatial and molecular precision in future photodynamic therapy designs. Future studies will focus on evaluating the *in vivo* therapeutic efficacy and biosafety profiles of this system to further assess its translational potential.

## Supplementary Material



## Data Availability

The data supporting this article are included in the Supporting Information.

## Notes

Experimental procedures; synthesis and characterization data for compounds 1–4 and **LAP-RI**; HPLC analysis; photophysical characterization; cell culture and treatments, cytotoxicity, ROS generation analysis, apoptosis/necrosis assay, and qRT-PCR experimental details; and ^1^H/^13^C NMR and HRMS spectra (PDF)

## ⊥

Department of Chemistry, University of Zurich, Zurich CH-8057, Switzerland

## References

[1] Mattiuzzi C., Lippi G. (2019). Current cancer epidemiology. J. Epidemiol.
Global Health.

[2] Sloan A. E., Ahluwalia M. S., Valerio-Pascua J., Manjila S., Torchia M. G., Jones S. E. (2013). Results of the NeuroBlate system first-in-humans
phase I clinical trial for recurrent glioblastoma. J. Neurosurg..

[3] Koo Y. E. L., Reddy G. R., Bhojani M., Schneider R., Philbert M. A., Rehemtulla A. (2006). Brain cancer diagnosis
and therapy with nanoplatforms. Adv. Drug Delivery
Rev..

[4] Van
Tellingen O., Yetkin-Arik B., De Gooijer M. C., Wesseling P., Wurdinger T., De Vries H. E. (2015). Overcoming the blood-brain
tumor barrier for effective glioblastoma treatment. Drug Resistance Updates.

[5] Rutka J. T., Kuo J. S. (2004). Pediatric surgical neuro-oncology:
current best care
practices and strategies. J. Neurooncol..

[6] Mackay A., Burford A., Carvalho D., Izquierdo E., Fazal-Salom J., Taylor K. R. (2017). Integrated
Molecular
Meta-Analysis of 1,000 Pediatric High-Grade and Diffuse Intrinsic
Pontine Glioma. Cancer Cell.

[7] Quail D. F., Joyce J. A. (2017). The Microenvironmental Landscape
of Brain Tumors. Cancer Cell.

[8] Gilbert M. R., Dignam J. J., Armstrong T. S., Wefel J. S., Blumenthal D. T., Vogelbaum M. A. (2014). A Randomized Trial of Bevacizumab for Newly
Diagnosed Glioblastoma. N. Engl. J. Med..

[9] Ponzoni M., Bachetti T., Corrias M. V., Brignole C., Pastorino F., Calarco E. (2022). Recent
advances in the developmental origin of neuroblastoma:
an overview. J. Exp. Clin. Cancer Res..

[10] Qiu B., Matthay K. K. (2022). Advancing therapy
for neuroblastoma. Nat. Rev. Clin. Oncol..

[11] żebrowska U., Balwierz W., Wechowski J., Wieczorek A. (2024). Survival Benefit
of Myeloablative Therapy with Autologous Stem Cell Transplantation
in High-Risk Neuroblastoma: A Systematic Literature Review. Target Oncol..

[12] Philippova J., Shevchenko J., Sennikov S. (2024). GD2-targeting therapy:
a comparative
analysis of approaches and promising directions. Front. Immunol..

[13] Persaud N. V., Park J. A., Cheung N. K. V. (2024). High-Risk
Neuroblastoma Challenges
and Opportunities for Antibody-Based Cellular Immunotherapy. J. Clin. Med..

[14] Karaman O., Alkan G. A., Kizilenis C., Akgul C. C., Gunbas G. (2023). Xanthene dyes
for cancer imaging and treatment: A material odyssey. Coord. Chem. Rev..

[15] Correia J. H., Rodrigues J. A., Pimenta S., Dong T., Yang Z. (2021). Photodynamic
Therapy Review: Principles, Photosensitizers, Applications, and Future
Directions. Pharmaceutics.

[16] Huang Z. (2005). A Review of
Progress in Clinical Photodynamic Therapy. Technol.
Cancer Res. Treat.

[17] Dolmans D. E., Fukurmura D., Jain R. K. (2003). Photodynamic therapy
for cancer. Nat. Rev. Cancer.

[18] Lucky S. S., Soo K. C., Zhang Y. (2015). Nanoparticles in Photodynamic Therapy. Chem. Rev..

[19] Zhao X., Liu J., Fan J., Chao H., Peng X. (2021). Recent progress in
photosensitizers for overcoming the challenges of photodynamic therapy:
from molecular design to application. Chem.
Soc. Rev..

[20] Agostinis P., Berg K., Cengel K. A., Foster T. H., Girotti A. W., Gollnick S. O. (2011). Photodynamic therapy of cancer: An update. CA Cancer J. Clin..

[21] Lee C. N., Hsu R., Chen H., Wong T. W. (2020). Daylight Photodynamic Therapy: An
Update. Molecules.

[22] Dougherty T. J., Gomer C. J., Henderson B. W., Jori G., Kessel D., Korbelik M. (1998). REVIEW
Photodynamic Therapy. J. Natl. Cancer Inst..

[23] Castano A. P., Demidova T. N., Hamblin M. R. (2004). Mechanisms
in photodynamic therapy:
Part one - Photosensitizers, photochemistry and cellular localization. Photodiagn. Photodyn. Ther..

[24] Josefsen L. B., Boyle R. W. (2008). Photodynamic therapy:
Novel third-generation photosensitizers
one step closer?. Br. J. Pharmacol..

[25] Li X., Kolemen S., Yoon J., Akkaya E. U. (2017). Activatable Photosensitizers:
Agents for Selective Photodynamic Therapy. Adv.
Funct. Mater..

[26] Zhao X., Zhao K. C., Chen L. J., Liu Y. S., Liu J. L., Yan X. P. (2021). A pH reversibly
activatable NIR photothermal/photodynamic-in-one
agent integrated with renewable nanoimplants for image-guided precision
phototherapy. Chem. Sci..

[27] Gerweck2 L. E., Seetharaman K. (1996). Cellular pH
Gradient in Tumor versus Normal Tissue:
Potential Exploitation for the Treatment of Cancer. Cancer Res..

[28] Chan C. F., Zhou Y., Guo H., Zhang J., Jiang L., Chen W. (2016). pH-Dependent Cancer-Directed Photodynamic Therapy by
a Water-Soluble Graphitic-Phase Carbon Nitride–Porphyrin Nanoprobe. ChemPlusChem.

[29] Zou J., Wang P., Wang Y., Liu G., Zhang Y., Zhang Q. (2019). Penetration depth tunable BODIPY derivatives for pH
triggered enhanced photothermal/photodynamic synergistic therapy. Chem. Sci..

[30] Meng L. B., Zhang W., Li D., Li Y., Hu X. Y., Wang L., Li G. (2015). PH-Responsive supramolecular vesicles
assembled by water-soluble pillar[5]­arene and a BODIPY photosensitizer
for chemo-photodynamic dual therapy. Chem. Commun..

[31] Best Q. A., Sattenapally N., Dyer D. J., Scott C. N., McCarroll M. E. (2013). PH-dependent
Si-fluorescein hypochlorous acid fluorescent probe: Spirocycle ring-opening
and excess hypochlorous acid-induced chlorination. J. Am. Chem. Soc..

[32] Lo P. C., Chen J., Stefflova K., Warren M. S., Navab R., Bandarchi B. (2009). Photodynamic molecular beacon triggered by
fibroblast activation protein on cancer-associated fibroblasts for
diagnosis and treatment of epithelial cancers. J. Med. Chem..

[33] Lovell J. F., Liu T. W. B., Chen J., Zheng G. (2010). Activatable photosensitizers
for imaging and therapy. Chem. Rev..

[34] Majumdar P., Nomula R., Zhao J. (2014). Activatable
triplet photosensitizers:
Magic bullets for targeted photodynamic therapy. J. Mater. Chem. C Mater..

[35] Li X., Kim J., Yoon J., Chen X. (2017). Cancer-Associated, Stimuli-Driven,
Turn on Theranostics for Multimodality Imaging and Therapy. Adv. Mater..

[36] Bhaumik J., Weissleder R., McCarthy J. R. (2009). Synthesis and photophysical properties
of sulfonamidophenyl porphyrins as models for activatable photosensitizers. J. Org. Chem..

[37] Lai J., Shah B. P., Garfunkel E., Lee K. B. (2013). Versatile fluorescence
resonance energy transfer-based mesoporous silica nanoparticles for
real-time monitoring of drug release. ACS Nano.

[38] Lee M. H., Kim J. Y., Han J. H., Bhuniya S., Sessler J. L., Kang C., Kim J. S. (2012). Direct
fluorescence monitoring of
the delivery and cellular uptake of a cancer-targeted RGD peptide-appended
naphthalimide theragnostic prodrug. J. Am. Chem.
Soc..

[39] Niu L. Y., Guan Y. S., Chen Y. Z., Wu L. Z., Tung C. H., Yang Q. Z. (2012). BODIPY-based ratiometric
fluorescent sensor for highly
selective detection of glutathione over cysteine and homocysteine. J. Am. Chem. Soc..

[40] Xu K., Qiang M., Gao W., Su R., Li N., Gao Y. (2013). A near-infrared reversible fluorescent probe for real-time
imaging of redox status changes in vivo. Chem.
Sci..

[41] Chen J., Jarvi M., Lo P. C., Stefflova K., Wilson B. C., Zheng G. (2007). Using the singlet oxygen
scavenging
property of carotenoid in photodynamic molecular beacons to minimize
photodamage to non-targeted cells. Photochem.
Photobiol. Sci..

[42] Yogo T., Urano Y., Kamiya M., Sano K., Nagano T. (2010). Development
of enzyme-activated photosensitizer based on intramolecular electron
transfer. Bioorg. Med. Chem. Lett..

[43] Ichikawa Y., Kamiya M., Obata F., Miura M., Terai T., Komatsu T. (2014). Selective
Ablation of β-Galactosidase-Expressing
Cells with a Rationally Designed Activatable Photosensitizer. Angew. Chem..

[44] Wang B., Chen Z., Cen X., Liang Y., Tan L., Liang E. (2022). A highly selective and sensitive chemiluminescent probe
for leucine aminopeptidase detection in vitro, in vivo and in human
liver cancer tissue. Chem. Sci..

[45] Chen Y. (2020). Fluorescent
probes for detection and bioimaging of leucine aminopeptidase. Mater. Today Chem..

[46] Li Z.-J., Wang C.-Y., Xu L., Zhang Z.-Y., Tang Y.-H., Qin T.-Y., Wang Y. L. (2023). Recent Progress
of Activity-Based
Fluorescent Probes for Imaging Leucine Aminopeptidase. Biosensors (Basel).

[47] Ziemska J., Solecka J., Jarończyk M. (2020). In Silico Screening for Novel Leucine
Aminopeptidase Inhibitors with 3,4-Dihydroisoquinoline Scaffold. Molecules.

[48] Miettinen J. J., Kumari R., Traustadottir G. A., Huppunen M.-E., Sergeev P., Majumder M. M. (2021). Aminopeptidase
Expression in Multiple Myeloma
Associates with Disease Progression and Sensitivity to Melflufen. Cancers (Basel).

[49] Zhong R., Jiang R., Zeng J., Gong X., Yang X., He L. (2023). Enhancing the Selectivity of Leucine Aminopeptidase
Near-Infrared Fluorescent Probes for Assisting in Surgical Tumor Resection. Anal. Chem..

[50] Zhang H., Mao Z., Wang F., Yang G., Zhang Y., Zhang X. (2021). Multi-dimensional
imaging of endogenous leucine aminopeptidase via fast response fluorescent
read-out probe. Dyes Pigm..

[51] Li R., Guo J., Duan Y., Liu X., Gui L., Xu Y. (2022). Monitoring inflammation-cancer
progression by cell viscosity, polarity
and leucine aminopeptidase using multicolor fluorescent probe. Chem. Eng. J..

[52] Jin C., Yang L., Fang N., Li B., Zhu H.-L., Li Z. (2024). A novel near-infrared fluorescent probe for real-time monitoring
of leucine aminopeptidase activity and metastatic tumor progression. Talanta.

[53] Li L.-Y., Xiang F.-F., Zhang H., Wang D.-J., Fan L.-X., Jiang Z. (2024). Clinical fluorescence-guide
surgery: Tracking leucine
aminopeptidase for assisting head and neck squamous cell carcinoma
surgery via a near-infrared fluorescent probe. Sens. Actuators, B.

[54] Xu L., Ma M., Li J., Gao D., Ma P., Zhang F., Song D. (2023). Leucine Aminopeptidase-Mediated Multifunctional
Molecular Imaging
Tool for Diagnosis, Drug Evaluation, and Surgical Guidance of Liver-Related
Diseases. Anal. Chem..

[55] Du K., Sheng L., Luo X., Fan G., Shen D., Wu C., Shen R. (2021). A ratiometric fluorescent
probe based on quinoline
for monitoring and imaging of Leucine aminopeptidase in liver tumor
cells. Spectrochim. Acta, Part A.

[56] Jain A., Heremans I., Rademaker G., Detomasi T. C., Rohweder P., Anderson D. (2025). Leucine
aminopeptidase LyLAP enables lysosomal
degradation of membrane proteins. Science.

[57] Matsukawa T., Mizutani S., Matsumoto K., Kato Y., Yoshihara M., Kajiyama H., Shibata K. (2022). Placental
Leucine Aminopeptidase
as a Potential Specific Urine Biomarker for Invasive Ovarian Cancer. J. Clin Med..

[58] Alborzinia H., Flórez A. F., Kreth S., Brückner L. M., Yildiz U., Gartlgruber M. (2022). MYCN mediates cysteine
addiction and sensitizes neuroblastoma to ferroptosis. Nat. Cancer.

[59] Do L. K., Lee H. M., Ha Y.-S., Lee C.-H., Kim J. (2025). Amino acids
in cancer: Understanding metabolic plasticity and divergence for better
therapeutic approaches. Cell Rep.

[60] Schubert N. A., Chen C. Y., Rodríguez A., Koster J., Dowless M., Pfister S. M. (2022). Target
actionability review to evaluate CDK4/6
as a therapeutic target in paediatric solid and brain tumours. Eur. J. Cancer.

[61] Arslan B., Bilici K., Demirci G., Almammadov T., Khan M., Sennaroglu A. (2021). A leucine aminopeptidase
activatable photosensitizer for cancer cell selective photodynamic
therapy action. Dyes Pigm..

[62] Xu W., Wang J., Xu C., Hua J., Wang Y. (2021). A diketopyrrolopyrrole-based
ratiometric fluorescent probe for endogenous leucine aminopeptidase
detecting and imaging with specific phototoxicity in tumor cells. J. Mater. Chem. B.

[63] Zhou W., Liu Y., Ma Q., Feng G., Zhang J., Liu G. (2024). AIE-active lysosome-targeted fluorescent organic nanoparticles for
leucine aminopeptidase-activatable fluorescent imaging and precision
photodynamic therapy potential. Dyes Pigm..

[64] Bartusik-Aebisher D., Rogóż K., Henrykowska G., Aebisher D. (2026). Advances in Near-Infrared
BODIPY Photosensitizers: Design Strategies and Applications in Photodynamic
and Photothermal Therapy. Pharmaceuticals.

[65] Kwon N., Weng H., Rajora M. A., Zheng G. (2025). Activatable Photosensitizers:
From Fundamental Principles to Advanced Designs. Angew. Chem. Int. Ed.

[66] Bilici K., Cetin S., Celikbas E., Yagci Acar H., Kolemen S. (2021). Recent Advances in Cyanine-Based Phototherapy Agents. Front. Chem..

[67] Barut B., Barut E. N., Yalçın CÖ., Ali Y. A., Akkaya D., Seyhan G. (2024). The synthesis
and therapeutic effect of silicon­(IV)
phthalocyanines for colorectal cancer cells in photodynamic therapy
by altering Wnt/β-catenin and apoptotic signaling. J. Photochem. Photobiol., A.

[68] Almammadov T., Kolemen S. (2021). A hydrogen peroxide
responsive resorufin-based phototheranostic
agent for selective treatment of cancer cells. Dyes Pigm..

[69] Almammadov T., Atakan G., Leylek O., Ozcan G., Gunbas G., Kolemen S. (2020). Resorufin Enters the Photodynamic Therapy Arena: A
Monoamine Oxidase Activatable Agent for Selective Cytotoxicity. ACS Med. Chem. Lett..

[70] Almammadov T., Elmazoglu Z., Atakan G., Kepil D., Aykent G., Kolemen S., Gunbas G. (2022). Locked and Loaded: β-Galactosidase
Activated Photodynamic Therapy Agent Enables Selective Imaging and
Targeted Treatment of Glioblastoma Multiforme Cancer Cells. ACS Appl. Bio Mater..

[71] Jiang W., Liang M., Lei Q., Li G., Wu S. (2023). The Current
Status of Photodynamic Therapy in Cancer Treatment. Cancers.

[72] Wang X., Luo D., Basilion J. P. (2021). Photodynamic Therapy: Targeting Cancer Biomarkers for
the Treatment of Cancers. Cancers.

[73] Yan J., Liu H., Wu Y., Niu B., Deng X., Zhang L. (2023). Recent progress of self-immobilizing
and self-precipitating molecular
fluorescent probes for higher-spatial-resolution imaging. Biomaterials.

[74] Liu F., Ding X., Xu X., Wang F., Chu X., Jiang J. H. (2022). A Reactivity-Tunable
Self-Immolative Design Enables
Histone Deacetylase-Targeted Imaging and Prodrug Activation. Angew. Chem., Int. Ed..

[75] Huang H. Y., Fan S. Y., Chang E. H., Lam C. H., Lin Y. C., Lin X. H. (2020). Self-Immolative Difluorophenyl Ester Linker for Affinity-Based
Fluorescence Turn-on Protein Detection. Anal.
Chem..

[77] Rothe F., Patties I., Kortmann R.-D., Glasow A. (2022). Immunomodulatory Effects
by Photodynamic Treatment of Glioblastoma Cells In Vitro. Molecules.

[78] Hambsch P., Istomin Y. P., Tzerkovsky D. A., Patties I., Neuhaus J., Kortmann R.-D. (2017). Efficient
cell death induction in human glioblastoma
cells by photodynamic treatment with Tetrahydroporphyrin-Tetratosylat
(THPTS) and ionizing irradiation. Oncotarget.

[79] Jamali Z., Hejazi S. M., Ebrahimi S. M., Moradi-Sardareh H., Paknejad M. (2018). Effects of LED-Based photodynamic
therapy using red
and blue lights, with natural hydrophobic photosensitizers on human
glioma cell line. Photodiagn. Photodyn. Ther..

[80] Xiong J., Chu J. C. H., Fong W.-P., Wong C. T. T., Ng D. K. P. (2022). Specific
Activation of Photosensitizer with Extrinsic Enzyme for Precisive
Photodynamic Therapy. J. Am. Chem. Soc..

[81] Alvarez N., Sevilla A. (2024). Current Advances in
Photodynamic Therapy (PDT) and
the Future Potential of PDT-Combinatorial Cancer Therapies. Int. J. Mol. Sci..

[82] Tam L. K. B., Chu J. C. H., He L., Yang C., Han K.-C., Cheung P. C. K. (2023). Enzyme-Responsive Double-Locked Photodynamic
Molecular Beacon for Targeted Photodynamic Anticancer Therapy. J. Am. Chem. Soc..

[83] Li Y., Li Y., He G., Li X., Ding R., Yan R. (2025). Activatable Enzymatic Nanoplatform Incorporated into Microneedle
Patch for Relieving Tumor Hypoxia Augmented Photodynamic Therapy. Adv. Mater..

[84] Wang Y., Xu Y., Song J., Liu X., Liu S., Yang N. (2024). Tumor Cell-Targeting
and Tumor Microenvironment–Responsive
Nanoplatforms for the Multimodal Imaging-Guided Photodynamic/Photothermal/Chemodynamic
Treatment of Cervical Cancer. Int. J. Nanomedicine.

