# Supplementary material for: Leucine Aminopeptidase-Activatable Photosensitizer Enables Enzyme-Responsive Photodynamic Therapy in Neuroblastoma
Source: ACS Omega. 2026 Jun 3;11(23):34168–77. doi: 10.1021/acsomega.6c01215 (PMC13280826; doi:10.1021/acsomega.6c01215)
Supplement: Supplementary file 1 [file ao6c01215_si_001.pdf]

Supporting Information

Leucine Aminopeptidase Activatable Photosensitizer Enables Enzyme-Responsive Photodynamic Therapy in Neuroblastoma

Osman Karaman[a], Dilay Kepil[a], Asena Sayin[a], Mehrdad Forough[a,b], Erva Ozkan[c], Zubeyir Elmazoglu[a,d], Gorkem Gunbas[a*]*

[a] Department of Chemistry, Middle East Technical University, 06800 Çankaya, Ankara, Türkiye
[b] R&D and Innovation Department, Nanografi Nanotechnology, METU Technopolis, 06531 Çankaya, Ankara, Türkiye
[c] Department of Biochemistry, Ankara Medipol University, 06050 Altındağ, Ankara, Türkiye
[d] Department of Pharmacology, Ankara Medipol University, 06050 Altındağ, Ankara, Türkiye

List of Figures

Figure S1 HPLC spectrum of **LAP-RI**. 6
Figure S2 ^{1}H NMR spectrum of compound **1** in $CDCl_3$. 10
Figure S3 ^{13}C NMR spectrum of compound **1** in $CDCl_3$. 10
Figure S4 ^{1}H NMR spectrum of compound **2** in $CDCl_3$. 11
Figure S5 ^{13}C NMR spectrum of compound **2** in $CDCl_3$. 11
Figure S6 ^{1}H NMR spectrum of compound **3** in $CDCl_3$. 12
Figure S7 ^{13}C NMR spectrum of compound **3** in $CDCl_3$. 12
Figure S8 ^{1}H NMR spectrum of compound **4** in $CDCl_3$. 13
Figure S9 ^{13}C NMR spectrum of compound **4** in $CDCl_3$. 13
Figure S10 ^{1}H NMR spectrum of **LAP-RI** in $CDCl_3$. 14
Figure S11 HRMS Spectrum of **LAP-RI**. 14

List of Tables

Table S1 HPLC retention times and peak areas of **LAP-RI** 6

General:

The starting materials and solvents were obtained from Sigma Aldrich, abcr, TCI, and Merck. Prior to use, solvents utilized for column chromatography, including hexane, ethyl acetate (EtOAc), and dichloromethane (DCM), were distilled over calcium chloride ($CaCl_2$). Unless otherwise noted, all reactions were carried out under a nitrogen atmosphere. Reaction solvents, such as diethyl ether, tetrahydrofuran (THF), DCM, toluene, and dimethylformamide (DMF), were used directly from an MBraun MBSPS5 solvent drying system. Reaction progress was monitored by thin-layer chromatography (TLC) on Merck Silica Gel 60 F254 plates, with visualization under ultraviolet (UV) light at 245 nm and 366 nm. Product purification was achieved by column chromatography using Merck Silica Gel (particle size: 0.040–0.063 mm, 230–400 mesh ASTM). Structural characterization of the synthesized compounds was performed using nuclear magnetic resonance (NMR) spectroscopy and high-resolution mass spectrometry (HRMS). Proton (^{1}H) and carbon (^{13}C) NMR spectra were recorded in deuterated solvents on a Bruker Avance III Ultrashield 400 MHz spectrometer, with chemical shifts reported in parts per million (ppm) relative to tetramethylsilane (TMS) as the internal standard. HRMS data for novel compounds were obtained using a Time-of-Flight (TOF) mass analyzer on a Waters Synapt MS system. Photophysical properties were evaluated through absorption and fluorescence spectroscopy. Absorption spectra were measured using a Jasco V-730 UV-vis spectrophotometer (Easton, MD, USA), while fluorescence emission spectra were recorded with a Cary Eclipse spectrofluorometer (Santa Clara, CA, USA) equipped with a Cary single-cell Peltier temperature controller (Agilent Technologies). All measurements were conducted at room temperature in 10 mm quartz cuvettes (3.5 mL, 111-QS, Hellma).

Synthesis:

Synthesis of compound **1**

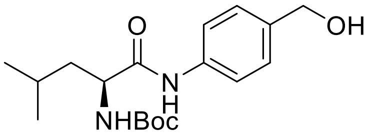

In a 50 mL two-necked round-bottom flask, commercially available N-(tert-butoxycarbonyl)-L-leucine (0.50 g, 2.0 mmol) and 4-aminobenzyl alcohol (0.12 g, 2.1 mmol, 1.1 eq.) were combined, followed by applying vacuum and nitrogen atmosphere. The mixture was dissolved in dry tetrahydrofuran (THF, 14 mL), after which N-ethoxycarbonyl-2-ethoxy-1,2-dihydroquinoline (EEDQ, 0.52 g, 2.1 mmol, 1.1 eq.) was added. The reaction was stirred at room temperature for 16 hours. Upon completion, THF was removed under reduced pressure, and the reaction mixture was quenched with distilled water (50 mL) before extraction with dichloromethane (DCM). The combined organic layers were dried over sodium sulfate (Na_2SO_4), filtered, and the solvent was evaporated. The crude product was purified by column chromatography on silica gel using methanol/DCM (5:95) as the eluent, yielding the target compound as a white solid (0.57 g, **85**%).^{1}H NMR (400 MHz, $CDCl_3$) δ 9.62 (s, 1H), 7.34 (d, *J* = 8.2 Hz, 2H), 6.99 (d, *J* = 8.1 Hz, 2H), 5.91 (s, 1H), 4.47 (s, 2H), 4.09 (s, 1H), 1.73 (ddd, *J* = 14.2, 10.9, 5.3 Hz, 2H), 1.60 (dd, *J* = 15.1, 6.5 Hz, 1H), 1.38 (s, 9H), 0.96 (d, *J* = 6.5 Hz, 3H), 0.93 (d, *J* = 6.4 Hz, 3H). ^{13}C NMR (100 MHz, $CDCl_3$) δ 172.4, 156.7, 137., 136.45, 127.5, 119.9, 80.2, 64.5, 54.0, 41.5, 28.4, 24.8, 23.2, 21.7.

Synthesis of compound **2**

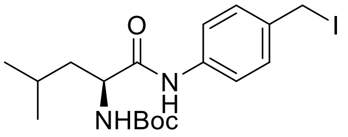

In a 50 mL Schlenk tube, triphenylphosphine (PPh_3, 0.61 g, 2.3 mmol, 1.5 eq.) and iodine (I_2, 0.60 g, 2.3 mmol, 1.5 eq.) were added, and the system was evacuated and purged with nitrogen. The mixture was dissolved in dry dichloromethane (DCM, 7 mL) and stirred at room temperature for 10 minutes. Imidazole (1.72 g, 1.1 mmol, 0.7 eq.) was then introduced, and the reaction was stirred for an additional 10 minutes. Compound **1** (0.52 g, 1.5 mmol) was subsequently added, and the mixture was stirred at room temperature for 2 days. Upon reaction completion, the mixture was quenched with distilled water (25 mL) and extracted with DCM. The combined organic layers were dried over sodium sulfate (Na_2SO_4), filtered, and the solvent was removed under reduced pressure. The crude product was purified by column chromatography on silica gel using methanol/DCM (3:97) as the eluent, affording compound **2** as a white solid (0.26 g, **37**%).^{1}H NMR (400 MHz, $CDCl_3$) δ 9.38 (s, 1H), 7.38 (d, *J* = 8.1 Hz, 2H), 7.17 (d, *J* = 7.9 Hz, 2H), 5.63 (s, 1H), 4.42 (s, 3H), 1.82 – 1.73 (m, *J* = 13.0, 6.5 Hz, 1H), 1.71 – 1.61 (m, *J* = 12.9, 6.9 Hz, 2H), 1.41 (s, 9H), 0.95 (dt, *J* = 11.4, 6.5 Hz, 6H). ^{13}C NMR (100 MHz, $CDCl_3$) δ 171.9, 156.7, 137.8, 134.7, 129.4, 120.1, 80.5, 54.1, 41.2, 28.5, 24.9, 23.2, 21.8.

Synthesis of compound **3**

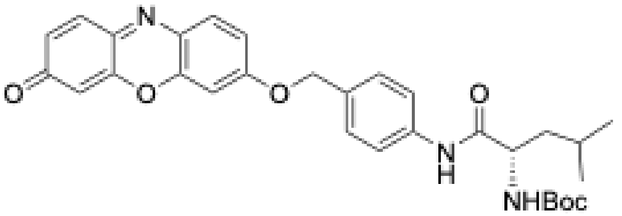

In a 25 mL Schlenk tube, compound **2** (70.0 mg, 0.20 mmol, 1.25 eq.) and commercially available resorufin sodium salt (26.4 mg, 0.12 mmol) were added, followed by evacuation and nitrogen backfilling. The mixture was dissolved in dry dimethylformamide (DMF, 5 mL), and potassium carbonate (K_2CO_3, 34.3 mg, 0.24 mmol, 2 eq.) was introduced. The resulting suspension was stirred at room temperature for 16 hours. Upon completion of the reaction, the mixture was quenched with distilled water (25 mL) and extracted with ethyl acetate (EtOAc, 50 mL). The combined organic layers were washed with brine (50 mL), dried over sodium sulfate (Na_2SO_4), filtered, and concentrated under reduced pressure. Purification of the crude product by column chromatography on silica gel using methanol/DCM (5:95) as the eluent yielded compound **3** as an orange solid (32.0 mg, **49**%).^{1}H NMR (400 MHz, $CDCl_3$) δ 8.69 (s, 1H), 7.69 (d, *J* = 8.9 Hz, 1H), 7.56 (d, *J* = 7.9 Hz, 2H), 7.41 (d, *J* = 9.8 Hz, 1H), 7.34 (d, *J* = 7.5 Hz, 2H), 6.98 (d, *J* = 10.5 Hz, 1H), 6.83 (d, *J* = 7.8 Hz, 2H), 6.31 (s, 1H), 5.09 (s, 2H), 5.05 (d, *J* = 7.8 Hz, 1H), 4.28 (s, 1H), 1.78 – 1.70 (m, 3H), 1.45 (s, 10H), 0.96 (t, *J* = 6.5 Hz, 7H). ^{13}C NMR (100 MHz, $CDCl_3$) δ 186.5, 171.1, 162.8, 156.5, 149.9, 145.8, 145.7, 138.4, 134.8, 134.4, 131.7, 131.1, 128.6, 128.5, 120.2, 114.4, 106.9, 101.2, 80.8, 70.7, 53.9, 40.5, 36.6, 31.6, 28.5, 24.9, 23.1, 22.1.

Synthesis of compound **4**

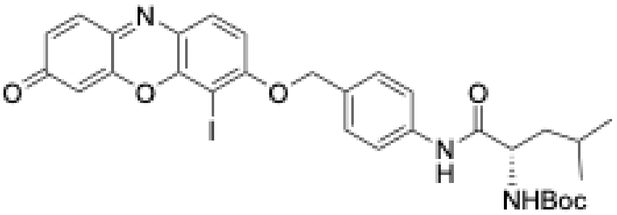

In a 25 mL Schlenk tube, compound **3** (32.0 mg, 0.06 mmol) was added, and the system was evacuated and purged with nitrogen. The compound was then dissolved in ethanol (EtOH, 7 mL). Molecular iodine (I_2, 38.2 mg, 0.15 mmol, 2.50 eq.) was added, and the reaction mixture was heated to 60 °C and stirred at that temperature for 10 minutes. Next, a solution of iodic acid (HIO_3, 5.30 mg, 0.03 mmol, 0.50 eq.) in water (0.50 mL) was added dropwise. Upon completion of the addition, the temperature was increased to 78 °C, and the mixture was stirred for an additional hour. Afterward, ethanol was evaporated, and the reaction was quenched with a 10% sodium thiosulfate ($Na_2S_2O_3$) solution (10 mL) and extracted with ethyl acetate (EtOAc). The combined organic layers were dried over sodium sulfate (Na_2SO_4), filtered, and concentrated under reduced pressure. The crude product was purified by column chromatography on silica gel using methanol/DCM (5:95) as the eluent, yielding the target compound as a dark orange solid (27.0 mg, **68**%).^{1}H NMR (400 MHz, $CDCl_3$) δ 8.95 (s, 1H), 7.73 (d, *J* = 8.8 Hz, 1H), 7.57 (d, *J* = 7.8 Hz, 2H), 7.41 (d, *J* = 9.7 Hz, 1H), 7.33 (d, *J* = 7.0 Hz, 2H), 7.02 (d, *J* = 9.7 Hz, 2H), 6.98 (s, 1H), 5.23 (d, *J* = 7.8 Hz, 1H), 5.10 (s, 2H), 4.37 (s, 1H), 1.80 – 1.74 (m, 2H), 1.68 – 1.61 (m, 1H), 1.47 (s, 9H), 0.98 (dt, *J* = 8.4, 6.1 Hz, 6H). ^{13}C NMR (100 MHz, $CDCl_3$) δ 180.5, 171.1, 163.0, 150.8, 145.7, 144.1, 138.2, 134.5, 131.9, 131.3, 130.5, 128.5, 128.3, 119.9, 119.6, 115.0, 100.9, 85.1, 80.6, 70.6, 53.8, 40.5, 29.6, 28.2, 24.7, 22.9, 21.7.

Synthesis of **LAP-RI**

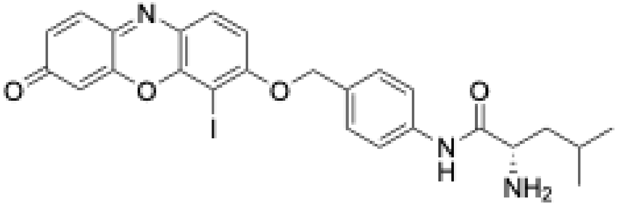

In a 25 mL Schlenk tube, compound **4** (27.0 mg, 0.05 mmol) was placed, and the system was evacuated and purged with nitrogen. The compound was then dissolved in dry dichloromethane (DCM, 5 mL). Trifluoroacetic acid (TFA, 0.30 mL) was added dropwise, and the resulting mixture was stirred at room temperature for 16 hours. Upon completion of the reaction, DCM and TFA were evaporated under reduced pressure. The crude product was purified by column chromatography on silica gel using methanol/DCM (5:95) as the eluent, yielding the target compound as a dark orange solid. For use in in vitro studies, **LAP-RI** was further purified by reverse-phase high-performance liquid chromatography (HPLC). ^{1}H NMR (400 MHz, $CDCl_3$) δ 9.66 (s, 1H), 7.76 (d, *J* = 9.5 Hz, 1H), 7.68 (d, *J* = 8.4 Hz, 2H), 7.48 – 7.37 (m, 3H), 7.09 – 7.04 (m, 2H), 7.03 (d, *J* = 9.7 Hz, 1H), 5.17 (s, 2H), 5.00 (d, *J* = 18.8 Hz, 1H), 1.49 – 1.39 (m, 2H), 1.35 (s, 1H), 0.98 (dt, *J* = 10.3, 6.2 Hz, 6H).

HPLC Analysis

Reverse phase HPLC analyses were conducted using Agilent Technologies 1260 Infinity II series HPLC systems with DAD detector. All the analyses were performed by using gradient elution with different Milli Q (0.1% TFA) and acetonitrile (0.8% TFA) systems as mobile phase and the column compartment temperature was 40°C. HPLC purifications were performed using a Phenomenex Kinetex 5 µm C18 100 A semi-preparative column with a flow rate of 1.5 mL/min. And for the purities, an Agilent Technologies Poroshell 120 EC-C18 analytical column was used with a flow rate of 0.5 ml/min.

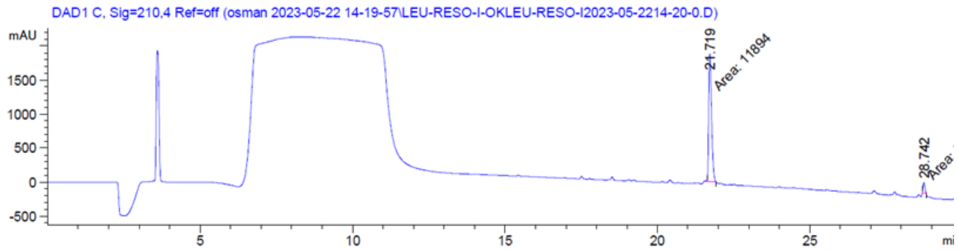

Figure S1 HPLC spectrum of **LAP-RI**.

Table S1 HPLC retention times and peak areas of **LAP-RI**

Peak	*Ret. Time (min)*	*Area (%)*
1	21.719	95.2642
2	22.83	4.7358

Photophysical Characterization

Materials:

The JASCO V-730 UV-Vis spectrophotometer and the Agilent Technologies Cary Eclipse fluorescence spectrophotometer were used for precise measurements and spectral analysis. To evaluate the specificity of the leucineaminopeptidase (LAP)-responsive photodynamic therapy agent (**LAP-RI**) toward the LAP enzyme, a variety of biologically relevant compounds were utilized. These compounds, sodium iodide (NaI), potassium chloride (KCl), lithium fluoride (LiF), calcium chloride ($CaCl_2$), and sodium nitrite ($NaNO_2$), were sourced from Sigma-Aldrich. Additionally, glucose, sodium thiosulfate ($Na_2S_2O_3$), thiourea (H_2NCSNH_2), and hydrogen peroxide (H_2O_2) were purchased from Merck, while homocysteine was obtained from Chem-Impex. Deionized water served as the solvent, and phosphate-buffered saline (PBS) from Serana was used to dilute the compounds. The diluted solutions were incubated with **LAP-RI** to investigate its selectivity for LAP. The LAP enzyme was subsequently employed to assess its enzymatic activity after incubation with **LAP-RI**.

Effects of LAP Concentration on Absorbance Spectra of LAP-RI

A comprehensive study was conducted to investigate the effect of LAP enzyme concentration on the absorbance spectra of the LAP-responsive iodo-resorufin (**LAP-RI**). A series of LAP enzyme solutions at varying concentrations (0.3 unit/mL, 0.5 unit/mL, 3 unit/mL, and 5 unit/mL) was prepared by diluting a stock enzyme solution in phosphate-buffered saline (PBS) containing 10 μM **LAP-RI**. Absorbance measurements were recorded after 95 minutes of incubation with each enzyme concentration. A reference control sample consisting solely of 10 μM **LAP-RI** in PBS was used for comparison.

The absorbance spectra exhibited distinct peaks, particularly at 410 nm, 490 nm, and 580 nm, corresponding to different states of **LAP-RI**, including its caged and activated forms. Statistical methods were employed to analyze the relationship between absorbance changes at 580 nm and LAP enzyme concentration.

Time-Dependent Activation Analysis of LAP-RI via LAP Cleavage

A systematic series of experiments was conducted to investigate the time-dependent activation of the LAP-caged iodo-resorufin-based photosensitizer **LAP-RI** via enzymatic cleavage of the LAP cage. The **LAP-RI** solution was prepared at 10 μM in phosphate-buffered saline (PBS) to ensure consistency and reproducibility across experiments. Enzyme solutions at a concentration of 0.3 unit/mL were prepared by diluting the enzyme in PBS containing 10 μM **LAP-RI**.

The activation process of **LAP-RI** over time was monitored by recording spectral data. Absorbance spectra were measured using a UV-Vis spectrophotometer, scanning wavelengths from 350 nm to 800 nm. Spectral measurements were taken at 10-minute intervals, starting 15 minutes after incubation of **LAP-RI** with the LAP enzyme. This comprehensive time-course analysis provided dynamic insights into the evolving absorbance characteristics of **LAP-RI**.

Cell Culture and Treatments

Human neuroblastoma cells (SH-SY5Y) and healthy mouse fibroblast cells (L929) were cultured in high-glucose Dulbecco's Modified Eagle's Medium (DMEM) supplemented with 10% fetal bovine serum (FBS) and 1% antibiotic-antimycotic solution. For the photodynamic therapy study, cells were exposed to increasing concentrations of **LAP-RI** (0.1–10 μM) for 1h, 2h, 4h or 6h at dark, followed by 2 h of 595 nm LED light. The irradiance at the cell-culture plate was measured as 8.12 mW/ cm^2, corresponding to a calculated nominal fluence of approximately 58.3 J/cm^2. No spectral absorption correction was applied; therefore, the reported fluence represents nominal delivered fluence rather than absorbed or effective fluence. Subsequently, the cells were incubated for up to 24 hours at 37 °C. A parallel procedure was conducted to assess dark cytotoxicity, omitting the LED light exposure step. (n=6)

Cytotoxicity Assay

Cytotoxicity was assessed using SH-SY5Y and L929 cells seeded in 96-well plates containing high-glucose, phenol red-free DMEM. Following the photodynamic therapy (PDT) protocol as described earlier, cells were incubated in the dark for up to 24 hours before measuring cell viability. At the end of the incubation periods, a fresh medium containing 0.5 mg/mL MTT was added to each well and incubated at 37°C for 2 -4 hours. Formazan crystals formed were solubilized by adding 10% SDS in PBS containing 0.01 N HCl. Absorbance measurements were recorded at 490 nm and 570 nm using a Multiskan Sky Microplate Reader (Thermo Scientific, USA). Cell viability results were expressed as a percentage relative to control samples treated with 0.2% DMSO. IC50 values were determined using concentration-normalized response curves derived from non-linear regression analysis (GraphPad Prism 9.02, GraphPad Software Inc.) (n=6).

Time-Dependent Activation

SH-SY5Y cells were plated in 96-well plates and treated with 1 μM **LAP-RI** for varying incubation times (0.5, 1, 2, 4, 6, or 8 hours). After incubation periods, cells were washed with 1X PBS twice then, stained with Hoechst 33342 (0.5 μg/mL in serum free DMEM) for 20 minutes at 37°C. Cells were subsequently washed three times with 1X PBS and fixed with 4% paraformaldehyde at room temperature for 30 minutes. After additional washing steps, confocal images were captured using a Zeiss LSM 900 CLSM with excitation/emission wavelengths of 361/497 nm for Hoechst and corresponding settings for **LAP-RI** (n = 4).

Intracellular ROS Generation

SH-SY5Y cells were seeded into 96-well plates and pre-incubated for 2 h in the presence or absence of specific reactive oxygen species (ROS) scavengers, including N-acetyl-L-cysteine (NAC; 2.5 or 5 mM), mannitol (25 or 50 mM), sodium azide (NaN_3; 1 or 2.5 mM), and tiron (1 or 2.5 mM). Following pre-treatment, cells were treated with **LAP-RI** (IC_{50}, 1 h) and subsequently subjected to LED irradiation for 2 h. For evaluation of intracellular ROS generation with confocal microscopy, cells were washed twice and incubated with 2',7'-dichlorodihydrofluorescein diacetate (DCFH-DA; 20 μM) and Hoechst 33342 (1 μg/mL) in serum-free DMEM for 15-20 min. After three washes with 1XPBS, fluorescence images were acquired using a Zeiss LSM 900 confocal laser scanning microscope (10× objective). DCF fluorescence was detected using 488/535 nm (ex/em), while Hoechst fluorescence was recorded at 361/497 nm (ex/em) wavelengths. All experiments were performed in six independent replicates (10X, n = 6).

Apoptosis/Necrosis Assessment

Apoptotic and necrotic cell death was assessed in SH-SY5Y neuroblastoma cells seeded in 48-well plates using the same experimental protocol employed for the intracellular ROS generation assay. Following LED irradiation, cells were incubated at 37 °C for 15-20 min

and subsequently stained with acridine orange (AO; 2.5 µg/mL) and ethidium bromide (EtBr; 2.5 µg/mL) prepared in serum-free DMEM for an additional 20 min. Cells were then washed with 1XPBS, and fluorescence images were acquired 500/525 nm (ex/em) and 530/617 nm (ex/em) wavelengths for AO and EtBr dyes, respectively using a Zeiss LSM 900 CLSM (10X, n=6). Apoptotic status was determined based on AO/EtBr staining patterns and nuclear morphology, where viable cells were defined as AO-positive/EtBr-negative cells with uniform green nuclei, early apoptotic cells as AO-positive/EtBr-negative cells showing bright green chromatin condensation or fragmentation, and late apoptotic/necrotic cells as EtBr-positive cells exhibiting orange/red nuclear staining with loss of nuclear integrity.

qRT-PCR Analysis for LAP3

The relative mRNA expression of LAP3 was quantified using RT-qPCR. Total RNA was isolated from SH-SY5Y and L929 cells using TRIzol reagent (ABP Biosciences, USA), following the manufacturer's protocol. The purified RNA was subsequently reverse-transcribed into cDNA using High-Capacity cDNA Reverse Transcription Kit (Fisher Scientific, USA). For quantitative PCR analysis, 5 µL of each cDNA sample was combined with 10 µL SYBR Green Master Mix, 2 µL of each gene-specific forward and reverse primer, and 6 µL of nuclease-free water. Amplification was performed on a Bio-Rad CFX96 real-time PCR system (Germany). The thermal cycling parameters consisted of an initial denaturation step at 95 °C for 5 min, followed by 40 cycles of denaturation at 95 °C for 30 s and annealing/extension at 65 °C for 60 s, with a final step at 95 °C for 5 s. GAPDH served as the reference gene, and relative expression levels were calculated using the $2^{-\Delta\Delta Ct}$ method (n=3). The following primer sequences were used in the experiment. LAP3: Forward: GAA GAT GGC TGT GTC GGC AAA G, Reverse: TCA TTG GCT GGC GTC TCC ATC. GAPDH: Forward: GTC TCC TCT GAC TTC AAC AGC G, Reverse: ACC ACC CTG TTG CTG TAG CCA A

Statistical Analysis

All statistical analyses were performed using GraphPad Prism software (version 9.02). Data obtained from MTT viability assays and ROS scavenger experiments were analyzed using the Kruskal-Wallis test, followed by Dunn's multiple comparisons post hoc test. Quantitative real-time PCR (qRT-PCR) data were analyzed using the Student's t-test. Results were expressed as mean ± SD, and p values < 0.05 were considered statistically significant.

NMR Spectra

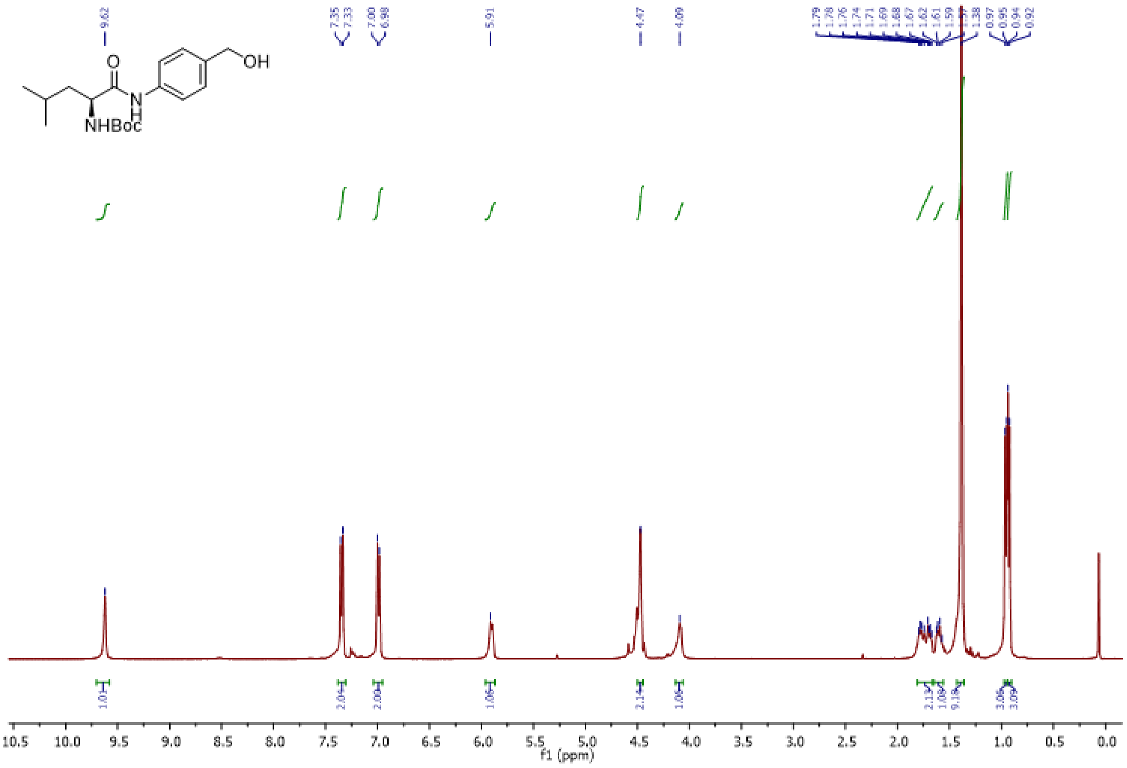

Figure S2 ^{1}H NMR spectrum of compound **1** in $CDCl_3$.

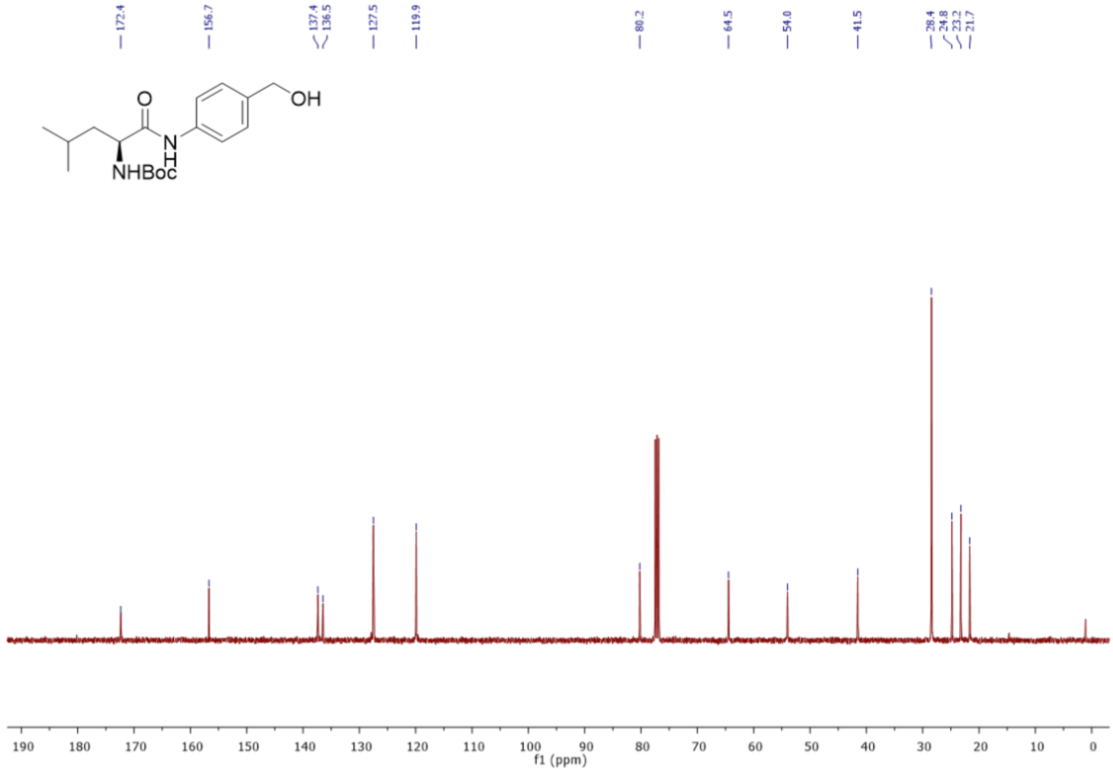

Figure S3 ^{13}C NMR spectrum of compound **1** in $CDCl_3$.

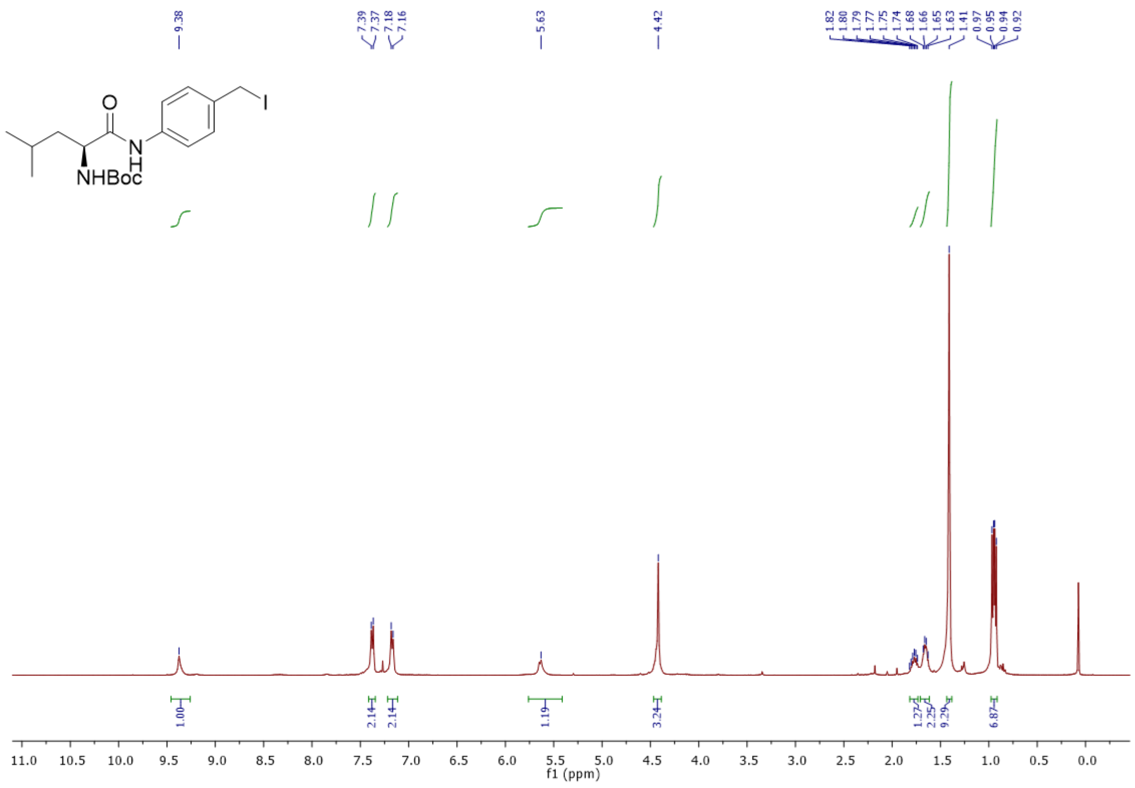

Figure S4 ^{1}H NMR spectrum of compound **2** in $CDCl_3$.

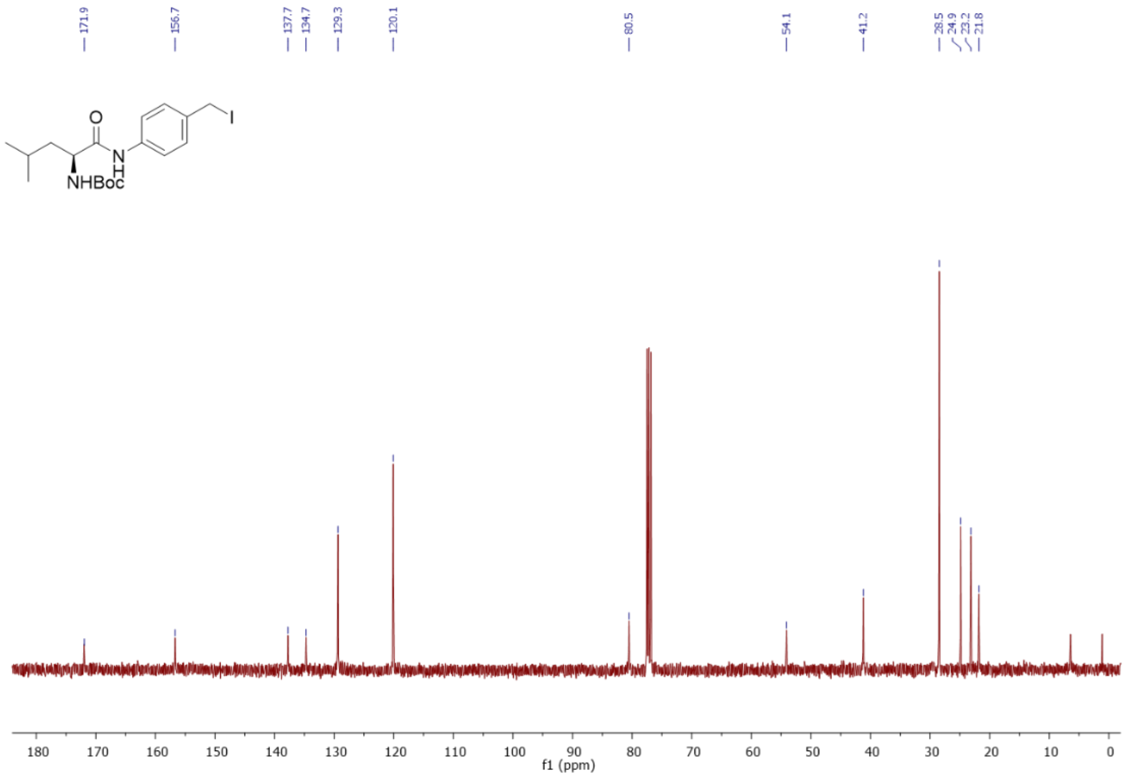

Figure S5 ^{13}C NMR spectrum of compound **2** in $CDCl_3$.

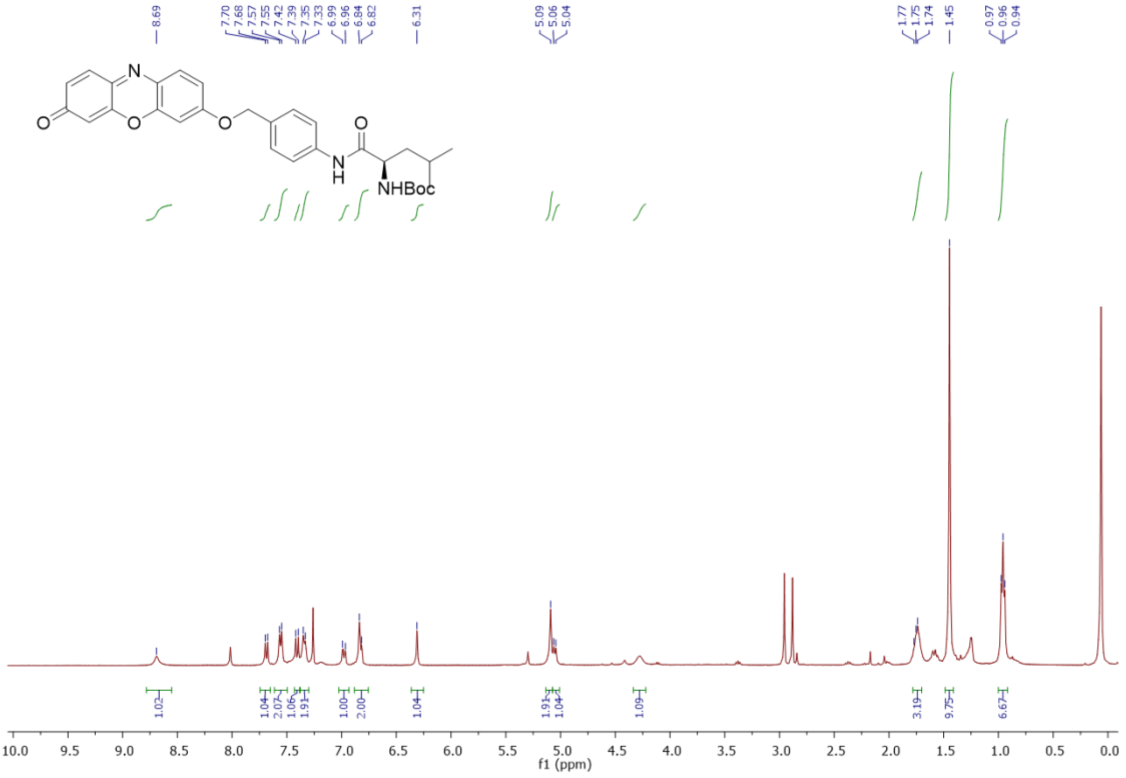

Figure S6 ^{1}H NMR spectrum of compound **3** in $CDCl_3$.

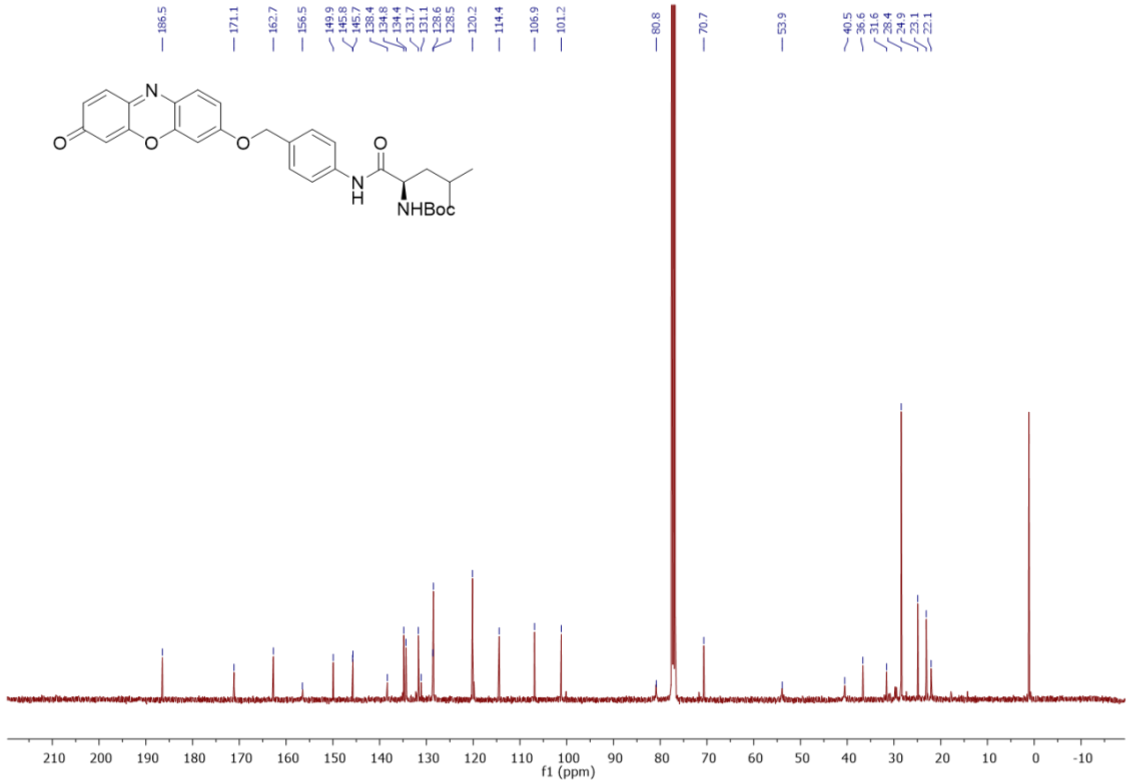

Figure S7 ^{13}C NMR spectrum of compound **3** in $CDCl_3$.

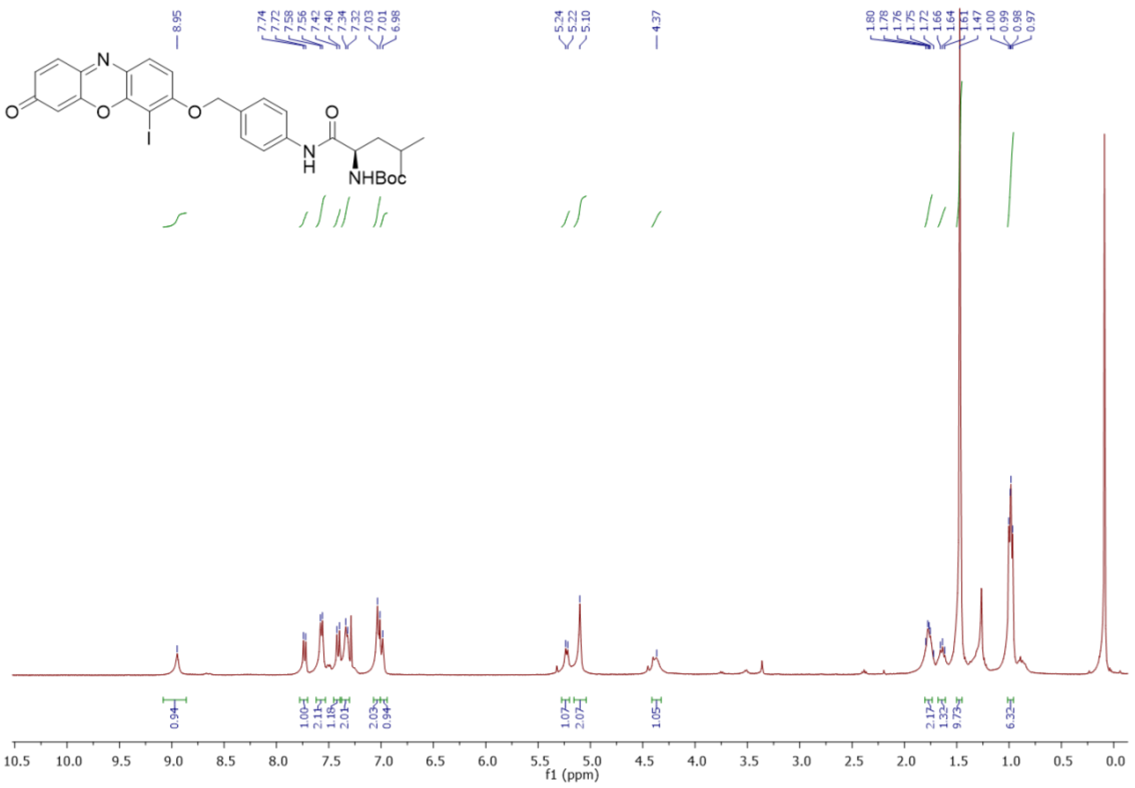

Figure S8 ^{1}H NMR spectrum of compound **4** in $CDCl_3$.

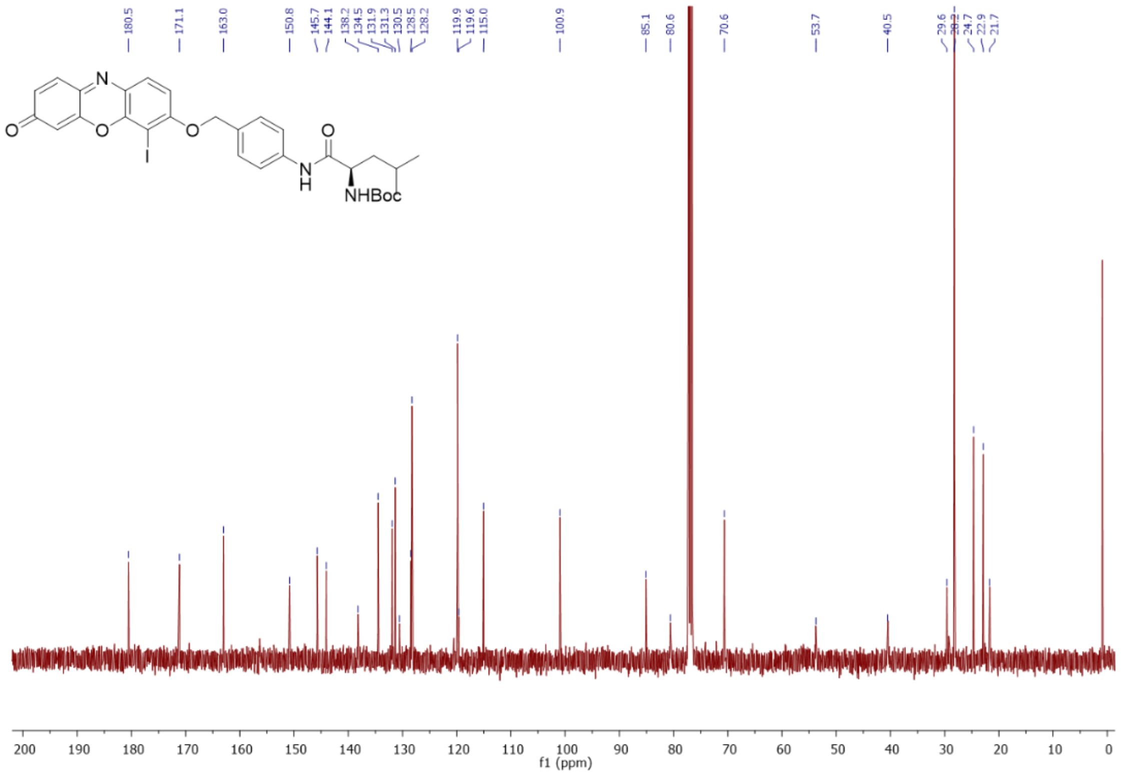

Figure S9 ^{13}C NMR spectrum of compound **4** in $CDCl_3$.

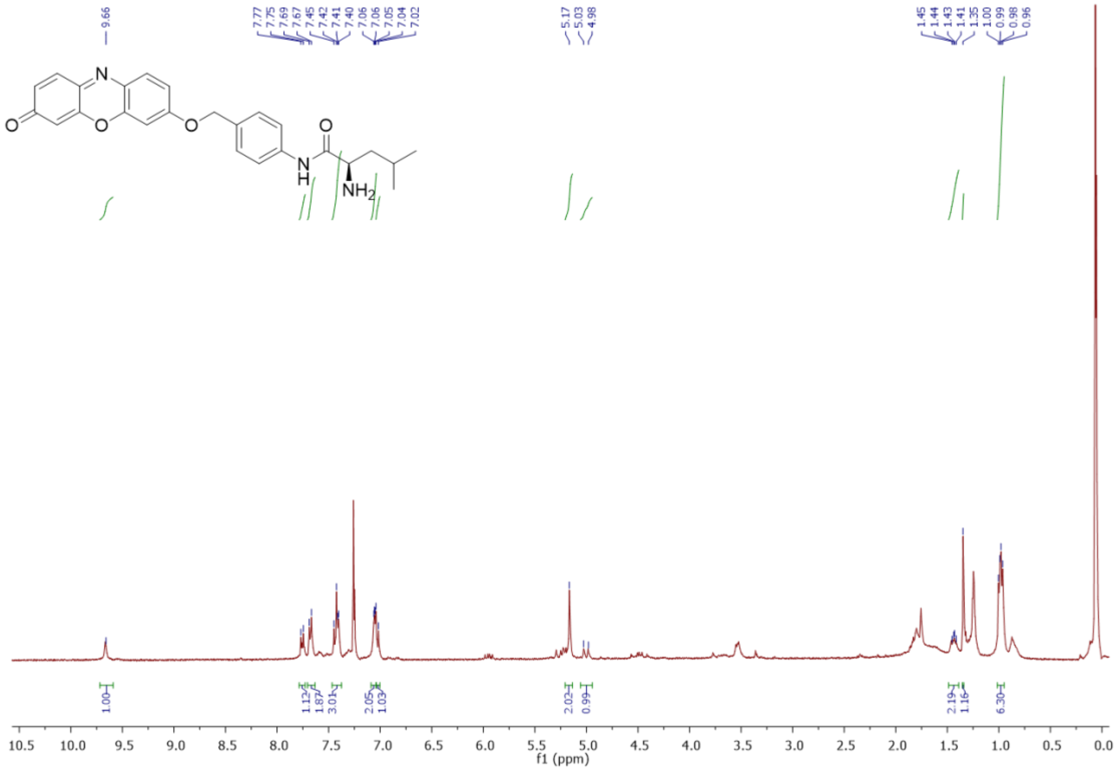

Figure S10 ^{1}H NMR spectrum of **LAP-RI** in $CDCl_3$.

HRMS Spectrum

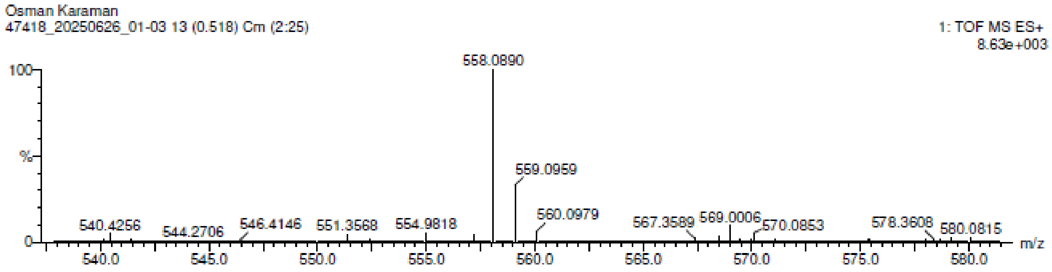

Minimum:				-1.5			
Maximum:		100.0	1000.0	50.0			
Mass	Calc. Mass	mDa	PPM	DBE	i-FIT	i-FIT (Norm)	Formula
558.0890	558.0890	0.0	0.0	14.5	263.1	0.0	C25 H25 N3 O4 I

Figure S11 HRMS Spectrum of **LAP-RI**.
